# Do behavioral drivers matter for healthcare decision-making during crises? A study of low-income women in El Salvador during the COVID-19 pandemic

**DOI:** 10.1186/s12889-024-19039-y

**Published:** 2024-08-06

**Authors:** Pedro Bernal Lara, Giuliana Daga, Lajos Kossuth, Florencia Lopez Boo

**Affiliations:** 1 Inter-American Development Bank, Social Protection and Health Division, 1300 New York Avenue NW, Washington, DC USA; 2 Sloan School of Management, Massachusetts Institute of Technology, 100 Main St, Cambridge, MA USA

**Keywords:** Healthcare decision-making, Behavioral economics, Low-income setting, Latin America, El Salvador

## Abstract

**Abstract:**

Understanding health-seeking behaviors and their drivers is key for governments to manage health policies. A growing body of research explores the role of cognitive biases and heuristics in health and care-seeking behaviors, but little is known about how a context of heightened anxiety and uncertainty might influence these behavioral drivers. This study analyzes the association between four behavioral predictors—internal locus of control, impatience, optimism bias, and aspirations—and healthcare decisions among low-income women in El Salvador, controlling for other factors. We find positive associations between internal locus of control and preventive health behaviors during the COVID-19 pandemic. For instance, a one standard deviation increase in locus of control is associated with a 10% increase in an index measuring the use of masks, distancing, hand washing, and vaccination. Locus of control was also associated with women’s use of preventive health services (one standard deviation improves the likelihood of having a hypertension test in the last six months by 5.8 percentage points). In a sub-sample of mothers, we find significant relationships between the four behavioral drivers and the decisions the mothers make for their children. However, we find these associations are less robust compared to the decisions they make for themselves. Some associations were stronger during the pandemic, suggesting that feelings of uncertainty and stress could amplify behavioral drivers’ influence on health-related behaviors. This novel finding is relevant for designing policy responses for future shocks.

**JEL Codes:**

I12, D10, D91, I30.

**Supplementary Information:**

The online version contains supplementary material available at 10.1186/s12889-024-19039-y.

## Introduction

Health-seeking decision-making is usually determined by need factors (such as chronic disease status or having a poor health perception) but also by key drivers such as education, health education, income, insurance status, and ability to pay for oneself [1]. However, healthcare decision-making is so complex that the typical neoclassical economics framework is not sufficient for analyzing it, and we might need to examine factors that go beyond observable variables [2, 3]. For instance, people might delay or avoid seeking preventive medical care in the present because they are myopic to the future gains of these interventions [4]. Others, by being overly optimistic, might underestimate their chances of contracting a disease that is actually seriously contagious [5].

Less is known about how factors that go beyond observable variables might influence healthcare decision-making in the context of a pandemic, an environment of heightened anxiety and uncertainty. Structural barriers like service disruption significantly influenced how much people utilized healthcare services. In four Latin American countries, service disruptions were associated with increased mortality rates [6], and in Mexico, they were associated with a decline of over 50% in sick child visits and with a 33% drop in consultations for childhood vaccinations [7]. However, data limitations made it difficult to disentangle the effect of supply and demand factors. In other developing areas where no restrictions were placed on healthcare services, like rural South Africa, a study found no change in adults’ total daily visits to clinics, but it did find a significant decrease in healthcare visits for children under one year old and one to five years old [8]. This finding resembles the decrease in children’s healthcare visits in China and children’s vaccination rates in the USA [9, 10]. Although the cause is unknown, insufficient childcare options for other children and the fact that these consultations rarely involve medication refills could explain this outcome. Finally, a study using a weekly country-by-county dataset covering the entire Taiwanese population finds that even without taking human mobility restrictions or supply constraints into account, people voluntarily reduced their demand for health care due to fears of contagion or to COVID-related prevention measures [11].

Researchers have paid less attention to how factors that go beyond structural or observable variables might influence healthcare decision-making during a pandemic. In this context, are people less rational, relying more on heuristics and their cognitive biases to make decisions? Or does the do-or-die nature of the context instead prompt them to prioritize “System 2 thinking,” [12] which is more rational and deliberate, but is slower and demands more cognitive resources. So far, the evidence on this topic seems mixed in low-income settings or when decisions are made for third parties (like when mothers make decisions on behalf of their children). Some studies have found no discernible differences in economic preferences and decision-making in times of crisis compared to normal times [13], while others report significant changes in risk tolerance and patience [14, 15], stress and depressive symptoms [16], and internet searches related to economic anxiety, mental health, and well-being [17–19]. The extent to which behavioral drivers impact decisions during crises could differ by gender. A study using an online sample of 1,500 respondents who were residents of the UK found that women had worse mental health than men and were more pessimistic about the pandemic and economic development [20].

This article thus explores how behavioral drivers affect healthcare decision-making during a pandemic in a low-income setting in a Central American country. We explore four types of behavioral drivers and their associations with the healthcare decisions of a cross-section of women (and with the healthcare decisions they made for their children in some cases) in low-income communities in El Salvador during the COVID-19 pandemic. Specifically, we rely on survey measures of impatience (how much people value the future versus the present), internal locus of control (the belief that one’s life is contingent on one’s own decisions), optimism bias (the belief that chances of positive events are higher for us than for our peers), and educational aspirations (for the women’s children) which relates to aspirations for the future. We test behavioral drivers’ predictive power by comparing healthcare decisions that were directly related to the COVID-19 pandemic and made during that time with decisions about general healthcare made one year later. The first set of decisions includes compliance with non-pharmaceutical COVID-19 prevention measures (masking, social distancing, and hand washing), COVID-19 vaccination, and avoidance of healthcare services for fear of the pandemic. In their econometric specification, we include restrictions in service supply during 2020 as controls. The second set of decisions includes preventive services for the women, such as screening for chronic conditions (hypertension and diabetes), as well as the use of health and nutrition services—such as prenatal care check-ups and feeding and supplementation practices— for their children.

Several key findings emerge from our analysis. First, locus of control is positively associated with multiple behaviors related to both COVID-19 and general health. For instance, the women’s internal locus of control is positively associated with non-pharmaceutical COVID-19 preventive measures, COVID-19 vaccination, and receiving preventive care services (hypertension check-ups). Interestingly, locus of control has the highest magnitude of correlation with daily behaviors that might be more challenging to adhere to, such as non-pharmaceutical COVID-19 preventive measures. This finding, which as far as we know has not been reported previously in the literature, is not surprising, since those who believe their fate is in their hands will likely take measures to avoid disease in the future.

Second, most behavioral drivers are relevant for non-pharmaceutical COVID-19 preventive measures. In this case impatience, locus of control, and optimism bias are all significant and have a meaningful magnitude of correlation. This is the only outcome for which all three behavioral drivers are jointly significant.

Third, the behavioral drivers of optimism bias and educational aspirations for children are relevant for health service avoidance during the pandemic. Women with higher optimism bias are less likely to have avoided health services for fear of the pandemic, perhaps because they were overconfident that they would not contract COVID-19 while receiving health services. In the same vein, mothers with higher educational aspirations for their children were less likely to avoid healthcare services for their children. This result is probably explained by a positive cost-benefit assessment of the risks of detecting children’s health needs and acting on time, even in the context of a pandemic.

Finally, in line with the literature, people with higher impatience are less likely to engage in healthy behaviors such as non-pharmaceutical COVID-19 prevention measures. These results provide evidence of the relevance of certain behavioral traits in healthcare decision-making during a crisis.

This article is structured as follows. The first section defines the behavioral predictors we use in this study and briefly reviews the literature on those predictors and their relationship with general, health-related decision-making. The second describes the data we used for our estimations. The third presents the econometric specifications and the main results, and the final section contains a brief discussion and a conclusion.

## Behavioral drivers and their relevance in the health literature

This section provides a brief overview of the behavioral predictors we use to study healthcare decision-making in the context of the COVID-19 pandemic in El Salvador. We define the predictors and contextualize them within the literature on healthcare decision-making.

### Internal locus of control

A person with a high internal locus of control believes that life events are contingent on their own decisions and behaviors instead of other forces like fate or luck [21]. Several studies have demonstrated a positive relationship between this attribute and different human capital investments, such as educational, job-seeking, and labor market decisions and outcomes [22–24]. In the health domain, the evidence shows that individuals with a higher internal locus of control prefer to be more present in the decision-making process and take a more active and collaborative role with doctors [25, 26]. Likewise, empirical research points to positive associations between higher locus of internal control and healthy living and well-being, and negative associations between this attribute and risky behavior such as tobacco, alcohol, and drug use [22, 27, 28]. People with a high internal locus of control also exhibit better physical and mental health and are less likely to suffer long-term health conditions [29]. In the field of prenatal and maternal health, a recent study in Nigeria shows that a higher internal locus of control was a significant predictor of utilization of antenatal care and skilled birth care, and of completion of child vaccination [30]. According to this evidence, people with a higher internal locus of control are more likely to display behaviors related to preventive health.

### Impatience

Impatience measures how much someone values the future relative to the present, and this preference affects inter-temporal decision-making. The literature contains ample evidence of the positive association between patience, human capital investments, and healthy lifestyles [31]. Further, impatience has been positively associated with obesity [32], and negatively with preventive health checkups such as fewer mammograms, Pap tests and prostate examinations, dental visits, and flu shot usage [4]. In sum, more impatient individuals are more likely to make less desirable health-related decisions, since most preventive health investments are realized in the future.

### Optimism bias

Optimism bias occurs when people think their chances of experiencing positive events are higher than that of their peers or of the public, or, conversely, that their chances of experiencing negative events are lower [33, 34]. A typical example is that most people claim they are less likely than the average driver to be involved in an automobile accident, which is mathematically unfeasible. This behavioral feature is also present in the health domain. People with optimism bias tend to believe they are less likely to experience negative health outcomes [35], which may hinder efforts to promote preventive, risk-reducing behaviors [36]. This type of bias is stronger among youth and among people with no active medical symptoms [37]. . In the context of the pandemic, this bias may help explain why many people refused to wear masks in health facilities and continued to attend large gatherings [38, 39]. However, evolutionary models suggest that individuals with optimism bias could be more likely to survive if the benefits of optimism outweigh the cost of inaccurately estimating risk [5, 40]. For example, there is a wide body of literature that finds that optimistic individuals were less likely to suffer blood pressure and cardiovascular disease because these people also had increased physical activity and better diet [41, 42].

### Aspirations

Parents’ aspirations about their children’s future play an important role in overall human capital investments. For example, mothers’ aspirations are important determinants of decisions about their daughters’ schooling [43] and of their children’s aspirations, achievements, and overall well-being [44]. Further, parental educational aspirations might lead adolescents to participate in health-promoting activities, like exercise or healthy eating [45]. According to this evidence, we expect the mothers in our sample with high educational aspirations for their children to behave in ways that promote their children’s health.

### Data

Our main data source is a household and facility survey conducted in June 2021 in low-income areas in El Salvador. At that time, the country was rolling out COVID-19 vaccinations, and about one in five people had received at least one dose. Meanwhile, the country was easing restrictions, and COVID-19 cases and mortality had stabilized after the Delta variant surge in January 2021.^1^ The main household survey respondents were women aged 15 to 49, and the questionnaire focused on utilization of health services for them and their children (for those with children younger than 60 months), as well as other health-related behaviors during the COVID-19 pandemic. The survey is unique in that it also includes measures on four behavioral predictors that could be relevant for healthcare decision-making in this context: impatience, internal locus of control, optimism bias, and mothers’ educational aspirations for their own children. In addition to the household survey, we administered a facility survey to the coordinator of the public primary care facility serving women in our sample.^2^ We merged the results from the facility survey with the results from the household survey. Overall, we collected information on 848 women and 60 facilities, which are representative of 14 of the poorest municipalities in El Salvador.^3^ We conducted sampling for the household survey in two stages. We first selected facilities within the 14 municipalities, followed by a random sample of dwellings in the facility’s catchment area to interview women. We interviewed women regardless of whether they received services from the facility.

### Main covariates

There are two categories of covariates in our dataset, as shown in Table 1. Panel A includes individual characteristics of the women and children, as well as proxies for their socioeconomic status. The women in our sample were around 31 years old on average. Most (68.28%) were either married or live in a de facto union, 27.95% have secondary or tertiary education, 57.43% perceive their health to be good, while only around 15% are first-time mothers, and 2.95% live in a household where someone had been diagnosed with COVID-19.^4^ The children in our sample were approximately 1.87 years old on average and were equally distributed by gender. Less than half of them (42.54%) were still breastfed, and their mothers were highly optimistic about their health status. Finally, we use housing conditions (for example, access to electricity, owning a bathroom, type of roof, and floor) and access to treated water as proxies for socioeconomic status. In our sample, only around 21% of households had three or more household assets (i.e., 79% of survey respondents had two or fewer assets), and only 34.67% had access to treated water, which underscores the low-income setting of the population in our sample.

**Table 1:** Descriptive statistics of covariates

	Mean	SD	No.
**Panel A. Household Survey**			
**Women**			
Age	30.89	8.67	848
Married / De facto union (0–1 = married or de facto union)	68.28%	0.47	848
Secondary or tertiary education (0–1 = secondary or tertiary education)d	27.95%	0.45	848
Self-reported good health (0–1 = good health)	57.43%	0.49	848
First-time mother (0–1 = first time mother)	15.09%	0.36	848
Woman or household member previously diagnosed with COVID-19	2.95%	0.17	848
**Children**			
Age	1.87	1.38	537
Female (0–1 = female)	50.09%	0.50	537
Mother’s confidence in children growing healthy and strong (0 = children not likely − 10= very likely )	9.08	1.47	537
Breastfed (0–1 = breastfed)	42.54%	0.49	536
**Household**			
Access to at least 3 assets in household (0 − 1 = 3 or more assets)	21.11%	0.41	848
Households that treat water (0–1 = treated water)	34.67%	0.48	848
**Panel B. Facility Survey Data** ^**1**^			
**Health services during 2020**			
Health service suspension (0–1 = if suspended during 2020)	44.34%	0.50	848
Suspension of child well-check visits (0–1 = if suspended during 2020)	16.16%	0.37	848
Staff reduction (0–1 = if the facility reduced staff during 2020)	35.26%	0.48	848
Reduction of business hours (0–1 = if the facility shortened business hours during 2020)	4.36%	0.20	848
Shortage of supplies (0 = no shortage − 10 = shortage of 10 elements)	1.88	1.93	848

*Note* Calculated by the authors based on survey data. The facility survey collects responses from coordinators of the main public primary care facility that women are assigned to. The responses from this survey were then merged with those of the household survey. Therefore, the statistics from Panel B reflect the status of these facilities as reported by their coordinator, but the percentages represent the women living in these facilities’ catchment area

Panel B describes how changes in health service provision during the pandemic affected the women and children in our sample. We find that just under 45% of women live in the catchment area of a facility that suspended health services at some point during 2020. However, child healthcare services were less affected, since only 16% of women experienced a suspension of services. Finally, even though over 35% of women lived in the catchment area of facilities that had staff reductions during 2020, only 4.36% experienced reduced hours. Overall, facilities serving these women had limited shortages of key supplies^5^ (with average shortages of just under two supplies).

### Explanatory variables: behavioral predictors

#### Locus of control

We use a survey measure of locus of control based on Caliendo et al. (2015) [46]. Table 2 details the survey questions used for this measure. We find that the population in our sample has an average internal locus of control score of 3.77 out of 5, with 5 being the highest degree of agreement with each statement. The standard deviation for this finding is 0.51. This score means the sample tends to agree more with statements that give themselves a higher degree of responsibility and agency over events. Although we did not find many differences across demographic groups, married women and those who completed high school education or higher have a slightly higher internal locus of control score (Table AS1). This finding is partially similar to data from Germany, where married individuals have a lower locus of control and higher educational attainment is associated with higher scores [46]. The same study also finds that women, immigrants, and older workers have a lower internal locus of control.

Comparing internal locus of control scores with other populations is challenging because of measurement invariance, especially considering the different interpretations of the construct and scales. However, the average in our sample is similar to the averages found in the literature. For instance, a study using a similar instrument to analyze a sample of unemployed individuals in Germany showed a slightly lower score of 3.58 [46].^6^ Other locus of control instruments implemented in household surveys in Australia [29] and Ethiopia [47] yielded averages that are relatively similar to ours when rescaled to a 5-point scale (3.92 for the former, and 3.45^7^ for the latter).

**Table 2:** Internal locus of control

Item No.	Components of internal locus of control	Mean	SD	Median
1	It is completely up to you what happens in your life	4.41	0.96	5.00
2	Compared to others, you have achieved what you deserve	4.06	1.09	4.00
3	What you achieve in your life depends, first, on fate or luck*	2.39	1.43	2.00
4	Most of the time, others make decisions about your life*	3.80	1.39	4.00
5	Success is obtained by working hard	4.77	0.64	5.00
6	When you encounter difficulties in your life, most of the time you doubt your abilities*	2.96	1.43	3.00
7	The opportunities you have in your life depend on the resources you have	4.20	1.05	5.00
8	Your effort is more important than your skills	4.23	1.03	5.00
9	You have little control over the things that happen in your life*	3.11	1.40	3.00
	**Internal locus of control (total score)**	**3.77**	**0.51**	**3.77**

*Note* Calculated by the authors based on survey data. We asked individuals the following question: “The following statements refer to attitudes towards life and the future. Please answer to what extent you agree with each of them on a scale of 1 to 5, where 1 is completely disagree and 5 is completely agree.” * We inverted the scores for Q3, Q4, Q6 and Q9 in this table so that the final index can be interpreted as internal locus of control

#### Impatience

We measure impatience according to Falk et al. (2022) [48]. We ask five questions using the staircase (or unfolding brackets) method, in which subjects choose between a payment today or a larger payment in twelve months. The amount of the hypothetical payment today is the same in each of the situations (10 USD), but the payment in 12 months increases if the respondent answers “today” and decreases if they answer “in 12 months.” All cases assume no inflation, so future prices will be the same as current prices. Each person is scored according to their preferences on a scale of 1 to 32, where 32 is high impatience (we provide a decision-tree with amounts in Appendix Figure AS1) We find a very skewed distribution, with a great majority of women in our sample reporting a high level of impatience: 78% of them answered they would prefer to receive 10 USD today, instead of 21.5 USD in 12 months.

We find significant temporal discounting levels, which aligns with previous related research, given the lower-income setting from which the sample was drawn [49].^8^ For larger and more heterogeneous populations, previous studies have found that patience is positively associated with higher cognitive ability and varies with age: middle-aged individuals are more patient than young and elderly people. However, we did not found differences by demographic variables (Table AS1), probably due to the homogeneity of the sample.

#### Optimism bias

Our survey module on optimism bias measures the women’s degree of confidence about future life events [33]. The survey questions used for this measure are detailed in Table 3. This indicator reflects the women’s estimate of how much their chances of experiencing five life events would differ from those of someone with similar characteristics, on a scale of 0 (not likely at all) to 10 (extremely likely). As with the locus of control scale, we inverted questions 2 and 5 to purely measure optimism bias. In general, we find the women in our sample to be overoptimistic. They tend to rate their own likelihood of experiencing a specific life event above the “equally as likely as my neighbor” cutoff for positive events (items 1, 3, and 4), and below and just around it for negative events (items 2 and 5). Concretely, they are optimistic about their economic situation (family having more income next year), and they think they are equally as likely to get robbed as their neighbors. This finding is in line with previous research on automobile accidents, crime, and disease [33, 51].

In the health domain, women seem to be relatively overconfident regarding their own health (living longer than 76 years, and *not* getting sick in the following months) and the health of their children (children growing up healthy and strong). This finding is also in line with the literature on health problems [36]. 9 In our sample, married women and women with children are more optimistic, especially with regards to health. In contrast, women with a high school education or higher are less optimistic about health than others with less education (Table AS1).

**Table 3:** Optimism bias

Item No.	Components of optimism bias	Mean	SD	Median
1	Live more than 76 years	5.52	3.01	5.00
2	That they rob me or someone in my family	5.12	3.35	5.00
3	That my family has more income next year	6.81	2.78	0.00
4	That in the next few months I get sick from something	3.93	2.81	5.00
5	That my children grow up strong and healthy	8.62	2.16	310
	**Total optimism bias**	**6.00**	**1.44**	**6.00**
	**Health-related optimism bias**	**6.02**	**1.62**	**6.33**

*Note* Calculated by the authors based on survey data. Individuals were asked the following question: “Below I will list events one by one. For each of them, I am going to ask you to tell me how likely you think it is that this event will happen to you in the future, compared to other women of similar age to yours in your community, where 0 is not at all likely, 5 is equally likely and 10 is very likely.” We invert questions 3 and 6 so that the final index can be interpreted as an optimism bias index

#### Educational aspirations

We measure educational aspirations as an indicator variable that takes a value of 1 if the mother aspires for their children to achieve a high school education or higher [52].^10^ The variable takes a value of 0 if she aspires for her children to complete secondary education or lower, and 1 if her aspirations are for them to partially complete tertiary education or higher. We find that around 73% would like their children to pursue more than a high school education. These percentages are higher than those reported in a rural and poor district in India, where 32% and 18% of parents want their male and female children to graduate from high school [52], but lower than the 81% of parents in the United States who report in a nationally representative survey in 2012 that they would like their children to complete superior education [53]. Within our sample, poorer households and women with lower levels of education had lower aspirations for their children (Table AS1).

In general, our population shows moderate levels of internal locus of control and high levels of impatience. Table 4 contains descriptive statistics for all our behavioral predictors. Our sample is overly optimistic about general future events, and even more so about health-related events. A majority of women want their children to pursue higher education, although this percentage is low compared to representative samples in industrialized countries. Table AS2 in the appendix shows the positive correlations between internal locus of control and educational aspirations, as well as a weaker correlation between internal locus of control and general optimism bias.

**Table 4:** Summary of behavioral drivers

	Mean	SD	*N*
Internal locus of control	3.77	0.51	848
Impatience	28.23	8.50	848
Total optimism bias	5.99	1.44	848
Health-related optimism bias	6.02	1.62	848
Educational aspirations	73.37%	0.44	537

*Note* Calculated by authors based on survey data. We measured internal locus of control on a scale of 1 to 5, where 5 is a high internal locus of control. The scale for measuring impatience was 1 to 32, where 32 is high impatience. We measured optimism bias on a scale of 0 to 10, where scores higher than five are more optimistic. Finally, we measured educational aspirations with a binary variable that takes a value of 1 if the mother aspires for her children to achieve more than a complete secondary education

### Outcome variables: healthcare decision-making

We classify the outcome variables in our study as either healthcare decision-making related to the COVID-19 pandemic, or what we consider to be general health behaviors (i.e., check-ups, preventive health services, and diet/nutrition, among others). The first category reflects novel behaviors that became relevant during the COVID-19 pandemic, whereas the second category contains usual health behaviors in the population of interest. Table 5 contains descriptive statistics for both types of behavior. In behaviors related to COVID-19 (Panel A of Table 5), 6.01% of the women in our sample report having avoided health services for themselves or a household member for fear of contracting COVID-19, while 8.33% of women with children under five years old avoided health services for their children because of that same reason. The average COVID-19 non-pharmaceutical prevention index for women in our sample was 3.05, which indicates that the women complied with at least three prevention measures (whether always using a mask, practicing social distancing, disinfecting their hands, or disinfecting objects around them in the last seven days). Finally, 83.84% of the women said they had been vaccinated against COVID-19 or were willing to be vaccinated.

**Table 5:** Descriptive data for outcome variables

	Mean	SD	*N*	
**Panel A. Health behaviors related to COVID-19**				
**Women**				
Avoidance of health services for self or household member for fear of contracting COVID-19	6.01%	0.24	848	
Number of non-pharmaceutical COVID-19 prevention measures undertaken (out of 4)	3.05	1.26	848	
% of women willing to get vaccinated or who already got vaccinated against COVID-19	83.84%	0.37	848	
**Children (0–5 years old)**				
Avoidance of health services for children for fear of contracting COVID-19	8.33%	0.27	518	
**Panel B. General health behaviors**				
**Women**				
Had blood pressure taken in the last 6 months (0–1 = had blood pressure taken)	30.31%	0.46	848	
Had a blood glucose level test in the last 6 months (0–1 = had test)	16.98%	0.38	848	
**Children (0–5 years old)**				
At least four prenatal visits (0–1 = at least four visits)	89.14%	0.31	534	
Percentage of children who had consumed micronutrients for more than 60 days in the last six months	10.45%	0.31	536	
Total number of iron-rich food items consumed by child in the last day (out of seven)	2.28	1.02	425*	

*Note* Calculated by the authors based on survey data. All percentage measures are binary variables that take the value of one if the described behavior exists. For iron-rich food items, we excluded children under the age of one because they usually have not started eating these food items at that age

Panel B of Table 5 shows that in our sample, around 30% of women had their blood pressure taken in the preceding six months and 17% had a blood glucose level test in that same period. This suggests relatively low take-up of these preventive services. Conversely, women with children under age five are extremely compliant with prenatal visits, as almost 90% of them report having gone to at least four during their most recent pregnancy in the last five years. Regarding feeding practices, only 10.45% of mothers gave their children the recommended dosage of micronutrients in the last six months, and, on average, they served only 2.28 out of 6 iron-rich food items in the last day.

### Econometric specification and results

To assess the relationship between our four behavioral predictors and the healthcare decisions people make in the context of the COVID-19 pandemic, we estimate a series of analogous OLS regressions. We split up the analysis according to the nature of outcomes. First, we focus on novel healthcare decisions related to the COVID-19 pandemic. Then we analyze general healthcare decisions that are independent of the pandemic (such as using preventive health services). The main specification for these regressions is:

$$\eqalign{{y_{if{\kern 1pt} j}} &= {\beta _0} + {\beta _1}\,Locu{s_{if{\kern 1pt} j}} + {\beta _2}\,Impatienc{e_{if{\kern 1pt} j}} \cr &+ {\beta _3}\,Optimism{\mkern 1mu} {\mkern 1mu} bia{s_{if{\kern 1pt} j}} + \delta {W_{if{\kern 1pt} j}} + \omega {H_{f{\kern 1pt} j}} + {\gamma _j} + {\varepsilon _{if\,j}} \cr}$$

(i) 

Where ${y}_{ifj}$ is one of our selected health outcomes for respondent *i* in facility *f* in municipality *j;*${Locus}_{ifj}$ is the internal locus of control measure for respondent *i* in facility *f* in municipality *j;*${Impatience}_{ifj}$ is the impatience measure for respondent *i* in facility *f* in municipality *j;*${Optimism bias}_{ifj}$ is the optimism bias measure at the individual level *i* in facility *f* in municipality *j;*${W}_{ifj}$ are individual controls for women; ${H}_{fj}$ are health services controls; ${\gamma }_{j}$ are municipality fixed effects; and ${\epsilon }_{ifj}$ is the error term. The coefficients of interest are ${\beta }_{1}$, ${\beta }_{2}$, and ${\beta }_{3}$, which measure the degree to which each behavioral predictor is associated with the specific health outcome. For child-specific outcomes, we take a subsample that includes only mothers and modify equation (i) by adding child individual controls, and include a fourth behavioral predictor: ${Aspirations}_{ifj}$. We use the same base specification for outcomes related to both COVID-19 and general health behaviors, except that we do not include health facility controls for general health behaviors since they have a different time frame: our health facility controls focus on how services were affected during 2020, whereas the reference period for general health behaviors is 2021. We conduct several robustness checks of the results for both sets of outcomes, assessing the individual association between each behavioral driver and outcome (Appendix Tables A3 and A4), sensitivity to different specifications (no controls and adding facility fixed effects), to multiple hypothesis testing, and to omitted variable bias (Appendix Tables AS5 and AS6), as well as to outliers (Appendix Figures AS2 and AS3).

### Results for COVID-19-related health behaviors

Our dataset contains the following COVID-19-related outcomes: (i) avoidance of health services (for the women, child, or another household member) for fear of the pandemic; (ii) compliance with COVID-19 non-pharmaceutical prevention measures; and (iii) having been vaccinated against COVID-19 or being willing to be vaccinated. All women in the sample responded the survey items on avoidance of health services for the woman or another household member, vaccination, and compliance with prevention methods. However, only women who are mothers of children between the ages of 0 and 5 responded to the question related to avoidance of health services for the child.

Table 6 presents the results of this first set of estimations. All behavioral predictors (impatience, internal locus of control, optimism bias, and educational aspirations) are standardized with a mean of 0 and standard deviation of 1 to make their association with the outcome variables comparable. We start with impatience. The literature predicts that more impatient individuals tend to adhere less to healthcare guidelines. Indeed, we find that an increase of one standard deviation in impatience is associated with 0.118 fewer points in an index of COVID-19 non-pharmaceutical prevention measures. Internal locus of control, on the other hand, is positively associated with COVID-19 non-pharmaceutical prevention measures, COVID-19 vaccination status, and healthcare avoidance: an increase of one standard deviation in internal locus of control predicts 0.277 more points in an index of COVID-19 preventive behaviors (a 10% increase relative to the mean), a 3.4 percentage points rise in the likelihood of having been vaccinated or being willing to be vaccinated, and a 2 percentage point increase in the likelihood of avoiding health services for fear of the pandemic. These results also align with the predictions in the literature: a higher degree of internal locus of control should be associated with believing that everyone is more in control of their destiny and thus that their health status is mainly their responsibility.

Optimism bias negatively predicts whether a woman or a household member avoided going to a healthcare facility for themselves or their child for fear of COVID-19. Our interpretation of these results is that women were overly optimistic about not contracting COVID-19 during their health visit. If they are biased towards optimism about remaining healthy despite the risk of contracting COVID-19, they might still decide to go to the health center and take care of their health needs. However, optimism bias is at the same time positively associated with compliance with non-pharmaceutical prevention measures. This surprising result might be related to different types of risks and behaviors in response to them. Finally, a mother’s educational aspirations for her children are negatively associated with avoiding health services for her children because of fear of COVID-19. We find that mothers with higher aspirations for their children are 3.2 percentage points less likely to avoid or postpone healthcare services for their children, which is in line with the literature. However, this result is not robust to the inclusion of facility fixed effects, as we lose precision, but the coefficient is still similar in magnitude (Appendix Table AS5).

**Table 6:** Behavioral predictors of health behaviors related to COVID-19

	Health behaviors related to COVID-19			
	Women			Children
	(1)	(2)	(3)	(4)
	**Women avoided health care for herself or someone in the household for fear of COVID-19**	**Women followed COVID-19 non-pharmaceutical prevention measures**	**Women got the COVID-19 vaccine or are willing to get it**	**Mothers avoided health care for their children for fear of COVID-19**
Impatience (z score)	0.001	-0.118***	0.005	0.008
	(0.010)	(0.039)	(0.013)	(0.015)
Internal locus of control (z score)	0.020**	0.277***	0.034**	-0.015
	(0.009)	(0.053)	(0.013)	(0.016)
Optimism bias (z score)	-0.031***	0.089*	0.000	-0.025
	(0.009)	(0.050)	(0.013)	(0.016)
Educational aspirations (z score)				-0.032**
				(0.016)
Individual controls	Yes	Yes	Yes	Yes
Household controls	Yes	Yes	Yes	Yes
Health services controls	Yes	Yes	Yes	Yes
Municipality FE	Yes	Yes	Yes	Yes
R-squared	0.059	0.138	0.058	0.114
Observations	848	848	848	518

Notes^*^p < 0.1, ^**^p < 0.05, ^***^p < 0.01. OLS estimations with robust standard errors clustered at the facility level in parentheses. Columns (1) to (3) present the results for the outcomes reported by women in the survey. Column (4) presents the results for child-related outcomes. Since only women with children report these outcomes, the sample size is smaller. The model for the outcomes for women follows equation (i) in section four and includes the individual, household, and facility controls listed in Table 1, as well as municipality fixed effects. The model for children in the last column follows the same equation but includes the child-level controls from Table 1 as well. The behavioral explanatory variables of interest are described as follows. Impatience is the standardized measure of the present bias index; internal locus of control is the standardized measure of the adapted locus of control index; optimism bias is the standardized measure of the general optimism bias index; and educational aspirations is the standardized measure of the educational aspirations for their children index

### Results for general health behaviors

We use the following general health outcomes as general health behaviors: (i) test for hypertension in the last six months; (ii) test for diabetes in the last six months; (iii) at least four prenatal visits; (iv) children’s consumption of micronutrients in the last six months; and (v) number of iron-rich food items consumed by children. Again, it is worth noting that the number of observations in each regression will depend on the type of outcome, as some apply to all women in the sample and others just to women with children. For example, the entire sample of women responded to the survey items on hypertension and diabetes. In contrast, only women who are mothers of children between the ages of 0 and 5 responded to the child-related outcomes, except for the question related to an iron-rich diet, which was restricted to mothers of children over one year old, where most children have made the transition to solid foods.

Table 7 presents the results of this second set of estimations. We focus on associations that are below the 5% significance level, as those significant at the 10% level are not robust (Appendix Table AS6). We observe that impatience negatively predicts feeding children micronutrients on 60 days in a six-month period. Internal locus of control, on the other hand, is positively associated with having been tested for hypertension in the last six months and an increased number of iron-rich food items consumed by children. Notably, an increase of one standard deviation in internal locus of control is associated with an increase of 5.8 percentage points in likelihood of having a hypertension screening. This correlation likely indicates that women who believe their destiny is in their own hands will try to guarantee the best possible health status. Although we find no significant association between internal locus of control and diabetes detection, this may be because the disease is less common than hypertension. Overall, Table 7 shows only weak associations for child-related general behaviors, as these associations are sensitive to our robustness checks (Appendix Table AS6), in contrast to the association between locus of control and hypertension screening, which is very strong. It is unclear whether the associations are weak because the behavioral predictors we measured for women do not have explanatory value for mothers’ caregiving behavior or because we have little power to detect them since the child-related sample is smaller. The associations we found were more robust in behaviors related to COVID-19, which suggests that feelings of uncertainty and stress could enhance the predictive power of our chosen behavioral predictor and that they may play an important role in novel behaviors.

**Table 7:** Behavioral predictors of general health behaviors

	General health behaviors				
	Women				Children
	(1)	(2)	(3)	(4)	(5)
	**Hypertension screening**	**Diabetes ** **screening**	**At least 4 prenatal care visits**	**Micronutrients adherence**	**Iron-rich diet**
Impatience (z score)	0.023*	0.015	-0.020*	-0.035**	-0.045
	(0.013)	(0.011)	(0.012)	(0.016)	(0.046)
Internal locus of control (z score)	0.058***	-0.008	0.028*	-0.007	0.124**
	(0.018)	(0.014)	(0.015)	(0.017)	(0.051)
Optimism bias (z score)	-0.009	0.012	-0.010	0.008	0.040
	(0.015)	(0.013)	(0.017)	(0.015)	(0.055)
Educational aspirations (z score)			0.020*	0.019*	0.062
			(0.011)	(0.011)	(0.053)
Individual controls	Yes	Yes	Yes	Yes	Yes
Household controls	Yes	Yes	Yes	Yes	Yes
Health services controls	No	No	No	No	No
Municipality FE	Yes	Yes	Yes	Yes	Yes
R-squared	0.071	0.061	0.133	0.136	0.181
Observations	848	848	534	536	425

Notes^*^p < 0.1, ^**^p < 0.05, ^***^p < 0.01. OLS estimations with robust standard errors clustered at the facility level in parentheses. Columns (1) and (2) present the results for the outcomes reported by women in the survey. Columns (3) to (5) present the results for child-related outcomes. Since only women with children report these outcomes, the sample size is smaller. The model for women outcomes follows Eq. (1) in section four and includes the women, household, and facility controls listed in Table 1, as well as municipality fixed effects. The model for the outcomes for women follows equation (i) in section four and includes the individual, household, and facility controls listed in Table 1, as well as municipality fixed effects. The model for children in the last column follows the same equation but includes the child-level controls from Table 1 as well. The behavioral explanatory variables of interest are described as follows. Impatience is the standardized measure of the present bias index; internal locus of control is the standardized measure of the adapted locus of control index; optimism bias is the standardized measure of the general optimism bias index; and educational aspirations is the standardized measure of the educational aspirations for their children index

## Conclusions

Historically, there have been clear socio-economic disparities in decision-making about the utilization of healthcare, which has always been shaped by need factors and observable drivers such as education, income, insurance status, and ability to pay. This study goes beyond traditional determinants and analyzes four types of behavioral predictors—impatience, internal locus of control, optimism bias, and aspirations—and their associations with decisions about healthcare among low-income women in El Salvador in the context of the COVID-19 pandemic.

Our results provide some novel insights. First, we find that our behavioral predictors are more significantly associated with healthcare decisions that are related to the pandemic. For example, impatience and locus of control have higher magnitudes and significance for COVID-19 prevention measurements. Especially salient is the case of optimism bias, which seems to predict less avoidance of health services for fear of the pandemic but exhibits no significant correlations with other decisions related to utilizing general health services. It is possible that the scale and nature of the event enhanced the influence of behavioral predictors on healthcare decisions to the detriment of a more rational approach to decision-making. This hypothesis is worth considering for future shocks like natural disasters, health emergencies, or situations of social unrest.

Second, most of the correlations we found align with what we would have predicted based on theory. Still, additional evidence is needed to support these conclusions, especially in a low-income setting. For instance, resembling previous literature, our study finds that, on average, higher internal locus is associated with healthier behaviors. Likewise, our study finds that women with optimism bias were less likely to avoid attending health facilities for fear of COVID-19, signaling that they were overconfident that they would not contract the disease. Moreover, impatience negatively predicts prevention measures, which denotes the tension between present costs and future health benefits. While these findings are aligned with previous research, this is one of the first studies that analyze these relationships in a low-income setting. Finally, while the behavioral predictors are significant and robust for women’s health behaviors, they are not robust in the subsample of mothers with children. From the available evidence, it is unclear whether the behavioral traits we measured for women are not relevant for health behaviors for their children or just that they have less power to detect them since the sample is smaller.

Our study has some limitations. First, since it is an observational study, we cannot rule out the presence of reverse causality, which can potentially influence the observed relationships between variables. For instance, it is not clear whether having good health might influence some of the behavioral drivers we analyze or vice-versa. Second, the generalizability of our findings may be restricted due to the specific demographic composition of our sample, which is consists exclusively of women from low-income settings. Finally, our survey was conducted in mid-2021, when El Salvador was transitioning out of lockdowns and expanding its coverage of COVID-19 vaccinations. Since this was an atypical time, it is not clear whether our findings for general health behaviors will be the same a few years after the pandemic. These limitations underscore the need for further research using diverse methodologies and broader study populations to validate and extend our findings.

Understanding people’s reasoning processes as they make healthcare decisions is key to improving policy design. Our study aims to expand the evidence on this topic with data from disadvantaged women in a developing country. To the best of our knowledge, it is also among the first to compare healthcare decisions related to the COVID-19 pandemic with those related to general health behaviors. In addition, we contribute to the literature by analyzing how these behavioral predictors affect third parties: the children of some of the women in our sample. Our results emphasize the need for further research that can provide specific strategies informed by behavioral sciences to improve health seeking behaviors and establish causal associations, which is something this study is not able to do. Our findings also shed light on the potentially effective role of behavioral strategies in improving the healthcare-seeking behaviors of the most vulnerable populations, which may have different patterns of locus of control, impatience, optimism, and aspirations than the general population.

### Electronic supplementary material

Below is the link to the electronic supplementary material.

Supplementary Material 1

Supplementary Material 2

## Data Availability

The datasets supporting the conclusions of this article are available in the IDB Social Data repository (https://scldata.iadb.org). It is available from the corresponding author upon reasonable request.

## Footnotes

1 This statement is based on data compiled by the Inter-American Development in a COVID-19 Situational Update report for Latin American and the Caribbean Bank as of June 15, 2021. The report is available at http://www.iadb.org/document.cfm? id=EZSHARE-2024879176-650.

2 Public health facilities are the main provider in survey locations. For example, according to UNICEF’s Multiple Indicator Cluster Survey (2014), in 2014, the Ministry of Health provided around 93% of postnatal care services in rural areas, which are the main type of areas captured in our survey.

3 The survey is representative of women living in 14 municipalities in El Salvador: Chiltiupán, Tacuba, El Sauce, Sociedad, Ilobasco, Sensuntepeque, Monte San Juan, San Cristóbal, San Antonio Masahuat, Santa María Ostuma, Apastepeque, San Esteban Catarina, San Ildefonso, and Tecoluca.

4 This refers only to self-reported COVID-19 diagnoses of woman or household members by test or health personnel prior to the survey. If someone in a household had COVID-19 at the time of the survey, that household was not included in the sample due to the health security protocol.

5 Examples of medical supplies include oral hydration salts, zinc, antibiotics for respiratory infections and vaccines (BCG, polio, measles, mumps, rubella, rotavirus, pneumococcal conjugate, or influenza).

6 Author’s calculation based on the procedure described in the footnote to Table 2 and by converting scores as a proportion of 5 instead of 7, which is the scale used for the study.

7 This score was calculated by reversing the items associated with external locus of control.

8 Previous research found that countries with lower incomes usually have greater temporal discounting [49], although economic inequality and broader financial circumstances are important [50].

9 It is possible that health-related optimism varies considerably depending on more specific health-related risks [35].

10 We ask mothers: “What is the highest level of completed education you would like for your child to achieve?”
