# Supplementary material for: Do behavioral drivers matter for healthcare decision-making during crises? A study of low-income women in El Salvador during the COVID-19 pandemic
Source: BMC Public Health. 2024 Aug 6;24:2122. doi: 10.1186/s12889-024-19039-y (PMC11302350; doi:10.1186/s12889-024-19039-y)
Supplement: Supplementary file 1 — Supplementary Material 1 [file 12889_2024_19039_MOESM1_ESM.pdf]

Appendix. Supplementary Tables and Figures

The Appendix contains additional descriptive characteristics of the behavioral drivers by demographic characteristics (Table A1), the correlation between behavioral predictors (Table A2), and an illustration of how we calculate our impatience measure (Figure A1).

It also includes several robustness checks to our main estimates. Tables A3 and A4, show whether our behavioral drivers are correlated individually with the variable of interest, rather than just between each other as when we include them jointly in our main estimates. The results show that the behavioral drivers are correlated individually with our variables of interest either with no controls or with the same controls we use in our main estimates. Tables A5 and A6 include specifications adding facility fixed effects, which will account for unobserved time invariant characteristics at the facility level, p-values accounting for multiple hypothesis testing, which are more conservative and account for the number of tests we are conducting in our main estimates, and Oster (2016) bounds which provide a bound on how sensitive our estimates could be to unobserved (omitted) variables. Finally in Figures A2 and A3 we test how sensitive our results are to outliers, by estimating our main model, but excluding one municipality at the time.

Table A1: Behavioral drivers' distribution by demographic characteristics

	(1)	(2)	(3)	(4)	(5)	(6)
	Mean		p-value	Mean		p-value
	Value=0	Value=1		Value=0	Value=1	
	Locus of control			Impatience		
Married / De facto union	3.71	3.80	0.00	-0.06	0.02	0.32
Secondary or Tertiary Education	3.73	3.88	0.03	0.01	-0.04	0.75
Self-reported good health	3.73	3.80	0.26	0.06	-0.06	0.74
Three or less household assets	3.79	3.70	0.44	0.00	-0.02	0.43
Woman has children	3.70	3.81	0.33	0.00	-0.01	0.73
	Optimism bias total			Optimism bias health		
Married / De facto union	5.89	6.05	0.10	5.82	6.11	0.06
Secondary or Tertiary Education	6.02	5.95	0.14	6.04	5.98	0.01
Self-reported good health	5.84	6.12	0.23	5.83	6.17	0.13
Three or less household assets	5.99	6.02	0.69	6.03	6.00	0.71
Woman has children	5.83	6.11	0.00	5.76	6.20	0.02
Total observations=848						
	Educational aspirations					
Married / De facto union	0.74	0.74	0.97			
Secondary or Tertiary Education	0.65	0.95	0.00			
Self-reported good health	0.70	0.76	0.14			
Three or less household assets	0.76	0.67	0.07			
Woman has children	-	0.73	-			
Total observations=507						

Notes: Columns (1) and (4) report the mean of the behavioral driver if the demographic variable is equal to 0; and Columns (2) and (5) report the mean when the demographic variable is equal to 1. Columns (3) and (6) report Pearson's chi squared test of the difference. The behavioral explanatory variables of interest are described as follows. Internal locus of control was measured on a scale from 1 to 5, where 5 is a high internal locus of control; impatience was measured on a scale from 1 to 32, being 32 with high impatience, its standardized version with mean 0 and standard deviation of 1 is reported; optimism bias was measured on a scale from 0 to 10 being scores higher than five more optimistic; finally, educational aspirations were measured with a binary variable that takes a value of 1 if the mother aspires their children to have an educational career such as medical doctors, lawyers, engineers, architects, or educators.

Table A2: Correlation Matrixes between behavioral predictors

Pearson's Correlations

Variables	(1)	(2)	(3)	(4)
(1) Optimism bias	1.000			
(2) Internal locus of control	0.067*	1.000		
(3) Present bias	-0.028	-0.061	1.000	
(4) Educational aspiration for offspring	0.072	0.117*	0.009	1.000

A. Correlation matrix of behavioral predictors using general optimism bias as one of the predictors. (*) denotes $p < 0.05$.

Pearson's Correlations

Variables	(1)	(2)	(3)	(4)
(1) Health-related optimism bias	1.000			
(2) Internal locus of control	0.039	1.000		
(3) Present bias	0.005	-0.061	1.000	
(4) Educational aspiration for offspring	0.068	0.117*	0.009	1.000

B. Correlation matrix of behavioral predictors using health-related optimism bias as one of the predictors. (*) denotes $p < 0.05$.

Table A3: Behavioral predictors of COVID-19-related health behaviors
Models including each behavioral predictor at a time

		Women						Children	
		**(1)**	**(2)**	**(3)**	**(4)**	**(5)**	**(6)**	**(7)**	**(8)**
		**Women avoided health care for herself or someone in the household for fear of COVID-19**		**Women followed COVID-19 non-pharmaceutical prevention measures**		**Women got the COVID-19 vaccine or is willing to get it**		**Mothers avoided health care for their children for fear of COVID-19**	
**Panel A. Impatience only**									
Impatience (z score)	Coef	-0.004	0.000	-0.145***	-0.133***	0.004	0.003	0.001	0.008
	Std. error	(0.009)	(0.009)	(0.040)	(0.038)	(0.014)	(0.014)	(0.013)	(0.013)
	R-squared	0.000	0.038	0.013	0.092	0.000	0.051	0.000	0.094
**Panel B. Locus of Control only**									
Internal locus of control (z score)	Coef	0.025***	0.018*	0.323***	0.288***	0.033**	0.034**	-0.021	-0.015
	Std. error	(0.009)	(0.010)	(0.043)	(0.043)	(0.013)	(0.014)	(0.014)	(0.014)
	R-squared	0.011	0.043	0.063	0.125	0.008	0.058	0.005	0.095
**Panel C. Optimism Bias only**									
Optimism bias (z score)	Coef	-0.026***	-0.030***	0.113***	0.104**	0.006	0.002	-0.026*	-0.024*
	Std. error	(0.009)	(0.009)	(0.042)	(0.044)	(0.013)	(0.013)	(0.013)	(0.014)
	R-squared	0.012	0.053	0.008	0.088	0.000	0.051	0.008	0.099
**Panel D. Aspirations only**									
Educational aspirations (z score)	Coef							-0.032**	-0.034**
	Std. error							(0.014)	(0.015)
	R-squared							0.013	0.105
Individual controls		No	Yes	No	Yes	No	Yes	No	Yes
Household controls		No	Yes	No	Yes	No	Yes	No	Yes
Health Services controls		No	Yes	No	Yes	No	Yes	No	Yes
Municipality fixed effects		No	Yes	No	Yes	No	Yes	No	Yes
Observations		848	848	848	848	848	848	528	518

Notes: * $p < 0.1$, ** $p < 0.05$, *** $p < 0.01$. OLS estimations with robust standard errors clustered at the facility level in parentheses. The model in each panel is that of the outcome of interest against just one behavioral explanatory variable at a time. Columns (1) to (6) present the results for outcomes reported by women in the survey. Columns (7) to (8) presents the results for children related outcomes. Since only women with children report these outcomes the sample size is smaller. The model for women outcomes that includes controls (columns 2, 4, and 6) follows equation (1) in section four and includes the women, household, and facility controls listed in Table 1, as well as municipality fixed effects. The model for children in the last column follows the same equation but includes the child level controls from Table 1 as well. The behavioral explanatory variables of interest are described as follows. Impatience is the standardized measure of the present bias index; internal locus of control is the standardized measure of the adapted locus of control index; optimism bias is the standardized measure of the general optimism bias Index; and educational aspirations is the standardized measure of the educational aspirations Index.

Table A4: Behavioral predictors of general health behaviors
Models including each behavioral predictor at a time

		Women				Children					
		(1)	(2)	(3)	(4)	(5)	(6)	(7)	(8)	(9)	(10)
		Hypertension screening		Diabetes screening		At least 4 prenatal care visits		Micronutrients adherence		Iron-rich diet	
**Panel A. Impatience only**											
Impatience (z score)	Coef	0.027*	0.020	0.022	0.015	-0.021*	-0.018	-0.030*	-0.035**	-0.031	-0.035
	Std. error	(0.015)	(0.015)	(0.012)	(0.012)	(0.012)	(0.012)	(0.017)	(0.017)	(0.039)	(0.044)
	R-squared	0.003	0.058	0.003	0.059	0.005	0.122	0.009	0.132	0.001	0.166
**Panel B. Locus of Control only**											
Internal locus of control (z score)	Coef	0.063***	0.056***	-0.006	-0.009	0.041***	0.029*	-0.016	-0.009	0.114**	0.118**
	Std. error	(0.016)	(0.017)	(0.013)	(0.013)	(0.016)	(0.016)	(0.015)	(0.015)	(0.046)	(0.048)
	R-squared	0.018	0.068	0.000	0.058	0.016	0.126	0.002	0.121	0.012	0.175
**Panel C. Optimism Bias only**											
Optimism bias (z score)	Coef	-0.008	-0.006	0.006	0.012	0.001	-0.012	0.010	0.009	0.047	0.030
	Std. error	(0.016)	(0.016)	(0.012)	(0.013)	(0.016)	(0.015)	(0.015)	(0.015)	(0.054)	(0.055)
	R-squared	0.000	0.056	0.000	0.059	0.000	0.120	0.001	0.121	0.002	0.165
**Panel D. Aspirations only**											
Educational aspirations (z score)	Coef					0.029*	0.021	0.012	0.018	0.085*	0.066
	Std. error					(0.015)	(0.015)	(0.013)	(0.013)	(0.049)	(0.051)
	R-squared					0.008	0.123	0.002	0.123	0.007	0.168
Individual controls		No	Yes	No	Yes	No	Yes	No	Yes	No	Yes
Household controls		No	Yes	No	Yes	No	Yes	No	Yes	No	Yes
Health Services controls		No	No	No	No	No	No	No	No	No	No
Municipality fixed effects		No	Yes	No	Yes	No	Yes	No	Yes	No	Yes
Observations		848	848	848	848	534	534	536	536	425	425

Notes: * $p < 0.1$, ** $p < 0.05$, *** $p < 0.01$. OLS estimations with robust standard errors clustered at the facility level in parentheses. The model in each panel is that of the outcome of interest against just one behavioral explanatory variable at a time. Columns (1) to (4) present the results for outcomes reported by women in the survey. Columns (5) to (10) presents the results for children related outcomes. Since only women with children report these outcomes the sample size is smaller. The model for women outcomes that includes controls (columns 2 and 4) follows equation (1) in section four and includes the women, household, and facility controls listed in Table 1, as well as municipality fixed effects. The model for children that includes controls (columns 6, 8, and 10) follows the same equation but includes the child level controls from Table 1 as well. The behavioral explanatory variables of interest are described as follows. Impatience is the standardized measure of the present bias index; internal locus of control is the standardized measure of the adapted locus of control index; optimism bias is the standardized measure of the general optimism bias Index; and educational aspirations is the standardized measure of the educational aspirations Index.

Table A5: Behavioral predictors of COVID-19-related health behaviors
Sensitivity to model specification, multiple hypothesis testing, and omitted variable bias

		Women									Children		
		(1)	(2)	(3)	(4)	(5)	(6)	(7)	(8)	(9)	(10)	(11)	(12)
		**Women avoided health care for herself or someone in the household for fear of COVID-19**			**Women followed COVID-19 non-pharmaceutical prevention measures**			**Women got the COVID-19 vaccine or is willing to get it**			**Mothers avoided health care for their children for fear of COVID-19**		
Impatience (z score)	Coef	0.001	-0.002	0.013	-0.118	-0.118	-0.073	0.005	0.007	0.006	0.008	0.001	-0.002
	Std.error.	(0.010)	(0.010)	(0.012)	(0.039)	(0.040)	(0.042)	(0.013)	(0.012)	(0.014)	(0.015)	(0.015)	(0.018)
	p-value	[0.936]	[0.806]	[0.270]	[0.004]	[0.004]	[0.087]	[0.695]	[0.569]	[0.662]	[0.592]	[0.941]	[0.921]
	Anderson (2008)	[0.493]	[0.368]	[0.217]	[0.009]	[0.007]	[0.096]	[0.425]	[0.323]	[0.495]			
	Romano-Wolf (2005)	[0.996]	[0.946]	[0.522]	[0.074]	[0.049]	[0.455]	[0.976]	[0.930]	[0.876]			
	List et al (2019))	[0.940]	[0.820]	[0.520]	[0.020]	[0.020]	[0.230]	[0.920]	[0.810]	[0.640]			
	Oster Coefficient Bounds												
	Coef (Rmax=1.3*R^2, δ=1)	0.002	-0.002	0.019	-0.108	-0.109	-0.046	0.005	0.008	0.007	0.009	0.001	-0.003
	Coef (Rmax=2.2*R^2, δ=1)	0.007	-0.001	0.039	-0.078	-0.080	0.055	0.006	0.010	0.009	0.012	0.001	-0.007
Internal locus of control (z score)	Coef	0.02	0.027	0.011	0.277	0.307	0.218	0.034	0.033	0.042	-0.015	-0.017	-0.016
	Std.error.	(0.009)	(0.008)	(0.011)	(0.053)	(0.056)	(0.058)	(0.013)	(0.013)	(0.015)	(0.016)	(0.015)	(0.018)
	p-value	[0.028]	[0.002]	[0.311]	[0.000]	[0.000]	[0.000]	[0.013]	[0.013]	[0.006]	[0.361]	[0.264]	[0.386]
	Anderson (2008)	[0.030]	[0.005]	[0.217]	[0.001]	[0.001]	[0.004]	[0.020]	[0.013]	[0.018]			
	Romano-Wolf (2005)	[0.187]	[0.018]	[0.602]	[0.000]	[0.000]	[0.002]	[0.129]	[0.075]	[0.053]			
	List et al (2019))	[0.040]	[0.010]	[0.300]	[0.000]	[0.000]	[0.000]	[0.020]	[0.010]	[0.010]			
	Oster Coefficient Bounds												
	Coef (Rmax=1.3*R^2, δ=1)	0.018	0.028	0.004	0.245	0.280	0.149	0.035	0.035	0.047	-0.011	-0.015	-0.013
	Coef (Rmax=2.2*R^2, δ=1)	0.009	0.032	-0.033	0.114	0.197	-0.226	0.037	0.040	0.074	0.005	-0.011	0.007
Optimism bias (z score)	Coef	-0.031	-0.028	-0.026	0.089	0.087	0.12	0.000	0.004	-0.003	-0.025	-0.023	-0.017
	Std.error.	(0.009)	(0.008)	(0.009)	(0.050)	(0.049)	(0.054)	(0.013)	(0.012)	(0.014)	(0.016)	(0.015)	(0.015)
	p-value	[0.001]	[0.001]	[0.006]	[0.081]	[0.079]	[0.029]	[0.990]	[0.750]	[0.853]	[0.129]	[0.145]	[0.260]
	Anderson (2008)	[0.003]	[0.004]	[0.018]	[0.058]	[0.056]	[0.046]	[0.493]	[0.368]	[0.611]			
	Romano-Wolf (2005)	[0.009]	[0.013]	[0.038]	[0.241]	[0.176]	[0.070]	[0.996]	[0.946]	[0.876]			
	List et al (2019))	[0.010]	[0.010]	[0.030]	[0.190]	[0.160]	[0.050]	[0.990]	[0.730]	[0.850]			
	Oster Coefficient Bounds												
	Coef (Rmax=1.3*R^2, δ=1)	-0.033	-0.029	-0.027	0.080	0.079	0.123	-0.002	0.003	-0.006	-0.024	-0.022	-0.013
	Coef (Rmax=2.2*R^2, δ=1)	-0.039	-0.033	-0.027	0.053	0.052	0.133	-0.008	0.001	-0.017	-0.022	-0.018	0.013
Educational aspirations (z score)	Coef										-0.032	-0.028	-0.027
	Std.error.										(0.016)	(0.014)	(0.017)

	Women									Children		
	(1)	(2)	(3)	(4)	(5)	(6)	(7)	(8)	(9)	(10)	(11)	(12)
	**Women avoided health care for herself or someone in the household for fear of COVID-19**			**Women followed COVID-19 non-pharmaceutical prevention measures**			**Women got the COVID-19 vaccine or is willing to get it**			**Mothers avoided health care for their children for fear of COVID-19**		
p-value										[0.046]	[0.047]	[0.117]
Oster Coefficient Bounds												
Coef (Rmax=1.3*R^2, δ=1)										-0.032	-0.025	-0.025
Coef (Rmax=2.2*R^2, δ=1)										-0.031	-0.018	-0.013

Continues on next page…

	(1)	(2)	(3)	(4)	(5)	(6)	(7)	(8)	(9)	(10)	(11)	(12)
Individual Controls	Yes	No	Yes	Yes	No	Yes	Yes	No	Yes	Yes	No	Yes
Household Controls	Yes	No	Yes	Yes	No	Yes	Yes	No	Yes	Yes	No	Yes
Health Services Controls	Yes	No	No	Yes	No	No	Yes	No	No	Yes	No	No
Municipality FE	Yes	No	No	Yes	No	No	Yes	No	No	Yes	No	No
Facility FE	No	No	Yes	No	No	Yes	No	No	Yes	No	No	Yes
Clusterred SE at facility level	Yes	Yes	Yes	Yes	Yes	Yes	Yes	Yes	Yes	Yes	Yes	Yes
R-squared	0.059	0.025	0.137	0.138	0.077	0.201	0.058	0.008	0.149	0.114	0.023	0.188
Observations	848	848	848	848	848	848	848	848	848	518	528	528

Notes: Columns (1) to (9) present the results for outcomes reported by women in the survey. Columns (10) to (12) presents the results for children related outcomes. Since only women with children report these outcomes the sample size is smaller. OLS estimations with robust standard errors clustered at the facility level in parentheses. P-values are presented in square brackets. The first p-value refers to the one obtained using the standard errors clustered at the facility level and that is equivalent to one of Tables 6 and 7 in the article. Below that are p-values adjusted for multiple hypothesis testing using different methodologies. For multiple hypothesis testing in this table, we group together all outcomes obtained from women (columns 1 to 9) using the same specification as a family of outcomes, and we consider children COVID-related behaviors as a separate family. Since the latter is just one outcome, we present no multiple hypothesis testing adjustment for this. Anderson (2008) refers to the q-value adjusted for false discovery rates; this is the least conservative adjustment. Romano-Wolf (2005) and List et al (2019) are both p-value adjustments for familywise error rates and both tend to be more conservative as they tend to control for reducing the likelihood of false positives. Oster coefficient bounds, refer to methods described in Oster (2016) to measure the stability of the coefficient of interest to the inclusion of unobserved variables, subject to some key assumptions regarding how much the R-squared will increase with the inclusion of such unobserved variables (Rmax) and the proportionality of the unobservable relative to the observable variables (δ). For this bound we use two scenarios presented in Oster (2019), one with Rmax=1.3*R^2 and δ=1, which is the most widely used in the literature and a more conservative one with Rmax=2.2*R^2 and δ=1. The result presented in the table is the bound to the coefficient of interest based on these assumptions if unobserved variables were included in the equation. Columns (1), (4), (7), and (10) present our preferred specification which we include in Tables 6 and 7 in the document. Columns (2), (5), (8) and (11) present the model without any controls. Columns (3), (6), (9) and (12) add facility fixed effects to the specification in addition to the individual and household controls. No facility controls or municipality fixed effects are included in this specification since they are collinear with the facility fixed effects. Section 4 in the paper describes the main specification and Table 1 includes the list of controls. The behavioral explanatory variables of interest are described as follows. Impatience is the standardized measure of the present bias index; internal locus of control is the standardized measure of the adapted locus of control index; optimism bias is the standardized measure of the general optimism bias Index; and educational aspirations is the standardized measure of the educational aspirations Index.

Table A6: Behavioral predictors of general health behaviors
Sensitivity to model specification, multiple hypothesis testing, and omitted variable bias

		Women						Children								
		(1)	(2)	(3)	(4)	(5)	(6)	(7)	(8)	(9)	(10)	(11)	(12)	(13)	(14)	(15)
		Hypertension screening			Diabetes			At least 4 prenatal care visits			Micronutrients adherence			Iron-rich diet		
Impatience (z score)	Coef	0.023	0.032	0.020	0.015	0.022	0.013	-0.020	-0.022	-0.013	-0.035	-0.030	-0.032	-0.045	-0.036	-0.082
	Std.error.	(0.013)	(0.014)	(0.015)	(0.011)	(0.011)	(0.012)	(0.012)	(0.012)	(0.013)	(0.016)	(0.014)	(0.017)	(0.046)	(0.038)	(0.055)
	p-value	[0.087]	[0.032]	[0.189]	[0.171]	[0.048]	[0.290]	[0.099]	[0.061]	[0.311]	[0.029]	[0.041]	[0.061]	[0.331]	[0.348]	[0.142]
	Anderson (2008)	[0.278]	[0.087]	[0.892]	[0.352]	[0.088]	[0.939]	[0.211]	[0.149]	[0.967]	[0.211]	[0.149]	[0.573]	[0.330]	[0.372]	[0.694]
	Romano-Wolf (2005)	[0.444]	[0.166]	[0.625]	[0.611]	[0.287]	[0.748]	[0.748]	[0.606]	[0.958]	[0.139]	[0.290]	[0.293]	[0.915]	[0.898]	[0.700]
	List et al (2019))	[0.170]	[0.080]	[0.320]	[0.170]	[0.050]	[0.270]	[0.200]	[0.170]	[0.290]	[0.080]	[0.110]	[0.130]	[0.340]	[0.360]	[0.240]
	Oster Coefficient Bounds															
	Coef (Rmax=1.3*R^2, δ=1)	0.022	0.034	0.018	0.012	0.021	0.01	-0.019	-0.022	-0.01	-0.037	-0.03	-0.033	-0.049	-0.037	-0.102
	Coef (Rmax=2.2*R^2, δ=1)	0.018	0.039	0.008	0.005	0.02	-0.003	-0.017	-0.023	0.002	-0.043	-0.03	-0.037	-0.065	-0.041	-0.185
Internal locus of control (z score)	Coef	0.058	0.066	0.071	-0.008	-0.005	0.002	0.028	0.039	0.015	-0.007	-0.018	-0.006	0.124	0.109	0.116
	Std.error.	(0.018)	(0.017)	(0.019)	(0.014)	(0.015)	(0.016)	(0.015)	(0.017)	(0.018)	(0.017)	(0.018)	(0.022)	(0.051)	(0.045)	(0.059)
	p-value	[0.002]	[0.000]	[0.000]	[0.540]	[0.742]	[0.917]	[0.066]	[0.022]	[0.409]	[0.655]	[0.328]	[0.786]	[0.019]	[0.017]	[0.054]
	Anderson (2008)	[0.013]	[0.002]	[0.003]	[0.521]	[0.591]	[1.000]	[0.211]	[0.149]	[0.967]	[0.488]	[0.372]	[1.000]	[0.211]	[0.149]	[0.573]
	Romano-Wolf (2005)	[0.003]	[0.001]	[0.001]	[0.787]	[0.839]	[0.918]	[0.466]	[0.115]	[0.958]	[0.944]	[0.785]	[0.980]	[0.219]	[0.290]	[0.485]
	List et al (2019))	[0.010]	[0.000]	[0.000]	[0.540]	[0.740]	[0.920]	[0.140]	[0.060]	[0.620]	[0.650]	[0.340]	[0.780]	[0.080]	[0.060]	[0.120]
	Oster Coefficient Bounds															
	Coef (Rmax=1.3*R^2, δ=1)	0.055	0.071	0.076	-0.009	-0.005	0.005	0.022	0.037	0.001	-0.004	-0.018	-0.001	0.128	0.106	0.117
	Coef (Rmax=2.2*R^2, δ=1)	0.046	0.085	0.105	-0.012	-0.003	0.025	-0.001	0.032	-0.084	0.01	-0.02	0.036	0.147	0.096	0.134
Optimism bias (z score)	Coef	-0.009	-0.012	-0.012	0.012	0.007	0.005	-0.010	-0.002	-0.008	0.008	0.009	0.007	0.040	0.041	0.038
	Std.error.	(0.015)	(0.015)	(0.016)	(0.013)	(0.012)	(0.013)	(0.017)	(0.018)	(0.018)	(0.015)	(0.013)	(0.017)	(0.055)	(0.054)	(0.062)
	p-value	[0.555]	[0.411]	[0.437]	[0.329]	[0.567]	[0.686]	[0.546]	[0.917]	[0.646]	[0.586]	[0.496]	[0.706]	[0.471]	[0.454]	[0.544]
	Anderson (2008)	[0.521]	[0.446]	[1.000]	[0.492]	[0.516]	[1.000]	[0.471]	[0.619]	[1.000]	[0.471]	[0.422]	[1.000]	[0.459]	[0.422]	[1.000]
	Romano-Wolf (2005)	[0.787]	[0.784]	[0.802]	[0.672]	[0.839]	[0.901]	[0.944]	[0.927]	[0.980]	[0.944]	[0.898]	[0.980]	[0.944]	[0.898]	[0.975]
	List et al (2019))	[0.560]	[0.600]	[0.610]	[0.520]	[0.570]	[0.680]	[0.810]	[0.920]	[0.860]	[0.590]	[0.750]	[0.690]	[0.840]	[0.840]	[0.870]
	Oster Coefficient Bounds															
	Coef (Rmax=1.3*R^2, δ=1)	-0.009	-0.014	-0.014	0.015	0.007	0.005	-0.015	-0.003	-0.012	0.007	0.009	0.005	0.037	0.039	0.034
	Coef (Rmax=2.2*R^2, δ=1)	-0.01	-0.018	-0.019	0.021	0.008	0.004	-0.033	-0.005	-0.038	0.005	0.009	-0.004	0.026	0.032	0.01
Educational aspirations (z score)	Coef							0.020	0.025	0.018	0.019	0.013	0.007	0.062	0.075	0.060
	Std.error.							(0.011)	(0.012)	(0.013)	(0.011)	(0.011)	(0.012)	(0.053)	(0.051)	(0.059)
	p-value							[0.078]	[0.045]	[0.164]	[0.099]	[0.221]	[0.567]	[0.250]	[0.148]	[0.317]
	Anderson (2008)							[0.211]	[0.149]	[0.694]	[0.211]	[0.284]	[1.000]	[0.273]	[0.209]	[0.967]
	Romano-Wolf (2005)							[0.748]	[0.489]	[0.904]	[0.772]	[0.858]	[0.980]	[0.827]	[0.687]	[0.929]
	List et al (2019))							[0.200]	[0.140]	[0.330]	[0.190]	[0.230]	[0.540]	[0.250]	[0.280]	[0.490]
	Oster Coefficient Bounds															
	Coef (Rmax=1.3*R^2, δ=1)							0.017	0.023	0.013	0.021	0.013	0.005	0.053	0.07	0.048
	Coef (Rmax=2.2*R^2, δ=1)							0.004	0.019	-0.011	0.031	0.014	-0.006	0.018	0.057	-0.017

Continues on next page…

	Women						Children								
	(1)	(2)	(3)	(4)	(5)	(6)	(7)	(8)	(9)	(10)	(11)	(12)	(13)	(14)	(15)
	Hypertension screening			Diabetes			At least 4 prenatal care visits			Micronutrients adherence			Iron-rich diet		
Individual Controls	Yes	No	Yes	Yes	No	Yes	Yes	No	Yes	Yes	No	Yes	Yes	No	Yes
Household Controls	Yes	No	Yes	Yes	No	Yes	Yes	No	Yes	Yes	No	Yes	Yes	No	Yes
Health Services Controls	No	No	No	No	No	No	No	No	No	No	No	No	No	No	No
Municipality FE	Yes	No	No	Yes	No	No	Yes	No	No	Yes	No	No	Yes	No	No
Facility FE	No	No	Yes	No	No	Yes	No	No	Yes	No	No	Yes	No	No	Yes
Clusterred SE at facility level	Yes	Yes	Yes	Yes	Yes	Yes	Yes	Yes	Yes	Yes	Yes	Yes	Yes	Yes	Yes
R-squared	0.071	0.023	0.13	0.061	0.004	0.126	0.133	0.027	0.22	0.136	0.062	0.232	0.181	0.091	0.289
Observations	848	848	848	848	848	848	534	534	534	536	536	536	425	425	425

Notes: Columns (1) to (6) present the results for outcomes reported by women in the survey. Columns (7) to (15) presents the results for children related outcomes. Since only women with children report these outcomes the sample size is smaller. OLS estimations with robust standard errors clustered at the facility level in parentheses. P-values are presented in square brackets. The first p-value refers to the one obtained using the standard errors clustered at the facility level and that is equivalent to one of Tables 6 and 7 in the article. Below that are p-values adjusted for multiple hypothesis testing using different methodologies. For multiple hypothesis testing in this table, we group together all outcomes obtained from women (columns 1 to 6) using the same specification as a family of outcomes, and we consider children general health behaviors as a separate family. Anderson (2008) refers to the q-value adjusted for false discovery rates; this is the least conservative adjustment. Romano-Wolf (2005) and List et al (2019) are both p-value adjustments for familywise error rates and both tend to be more conservative as they tend to control for reducing the likelihood of false positives. Oster coefficient bounds, refer to methods described in Oster (2016) to measure the stability of the coefficient of interest to the inclusion of unobserved variables, subject to some key assumptions regarding how much the R-squared will increase with the inclusion of such unobserved variables (Rmax) and the proportionality of the unobservable relative to the observable variables (δ). For this bound we use two scenarios presented in Oster (2019), one with Rmax=1.3*R^2 and δ=1, which is the most widely used in the literature and a more conservative one with Rmax=2.2*R^2 and δ=1. The result presented in the table is the bound to the coefficient of interest based on these assumptions if unobserved variables were included in the equation. Columns (1), (4), (7),(10) and (13) present our preferred specification which we include in Tables 6 and 7 in the document. Columns (2), (5), (8), (11), and (14) present the model without any controls. Columns (3), (6), (9), (12), and (15) add facility fixed effects to the specification in addition to the individual and household controls. No facility controls or municipality fixed effects are included in this specification since they are collinear with the facility fixed effects. Section 4 in the paper describes the main specification and Table 1 includes the list of controls. The behavioral explanatory variables of interest are described as follows. Impatience is the standardized measure of the present bias index; internal locus of control is the standardized measure of the adapted locus of control index; optimism bias is the standardized measure of the general optimism bias Index; and educational aspirations is the standardized measure of the educational aspirations Index.

Figure A1. Decision tree for the impatience measure

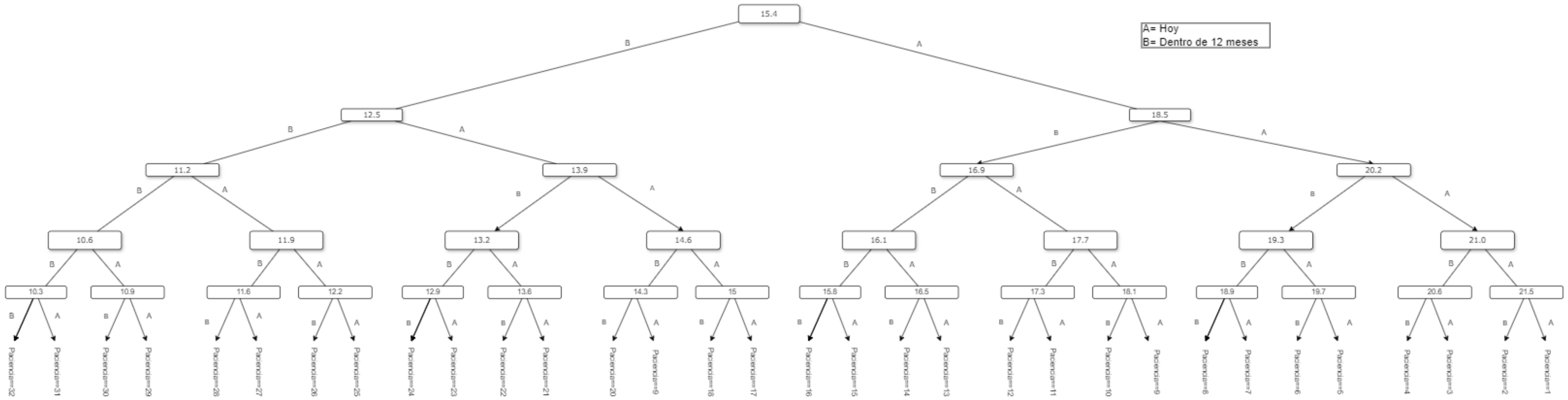

Notes: Decision tree for the impatience measure based on Falk et al. (2022). The amounts were updated to a similar amount to USD in El Salvador using purchasing power parity. A. Refers to payment today, and B for payment in 12 months. The impatience measure is the reverse of score since the tree was originally used to measure patience.

Figure A2. Sensitivity of the results of behavioral predictors of COVID-19-related health behaviors to municipal outliers.

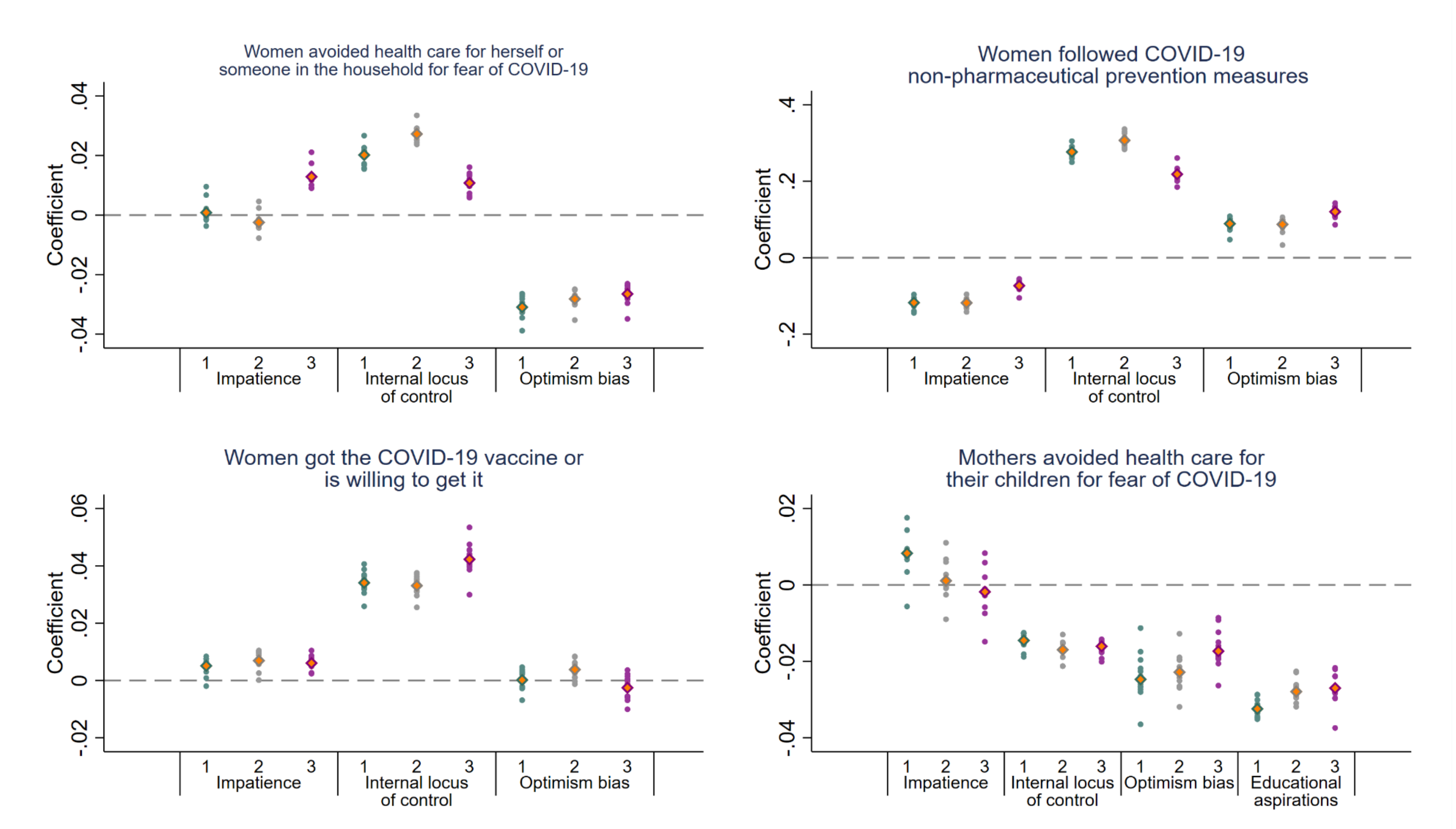

Notes: Each chart presents the coefficient of the behavioral predictors on our outcome of interest, which is presented on the title of each chart. The yellow diamond-shape presents the coefficient in a model with all 14 municipalities included. The dots represent the value of the coefficient of interest, excluding one of the 14 municipalities at a time. The green dots under over the value of 1 in the x-axis refer to our preferred specification which we include in Tables 6 and 7 in the document. The gray dots over the value of 2 in the x-axis, present the model without any controls. The purple dots over the value of 3 in the x-axis add facility fixed effects to the specification in addition to the individual and household controls. No facility controls or municipality fixed effects are included in this specification since they are collinear with the facility fixed effects. Section 4 in the paper describes the main specification and Table 1 includes the list of controls. The behavioral explanatory variables of interest are described as follows. Impatience is the standardized measure of the present bias index; internal locus of control is the standardized measure of the adapted locus of control index; optimism bias is the standardized measure of the general optimism bias Index; and educational aspirations is the standardized measure of the educational aspirations Index.

Figure A3. Sensitivity of the results of behavioral predictors of general health behaviors to municipal outliers.

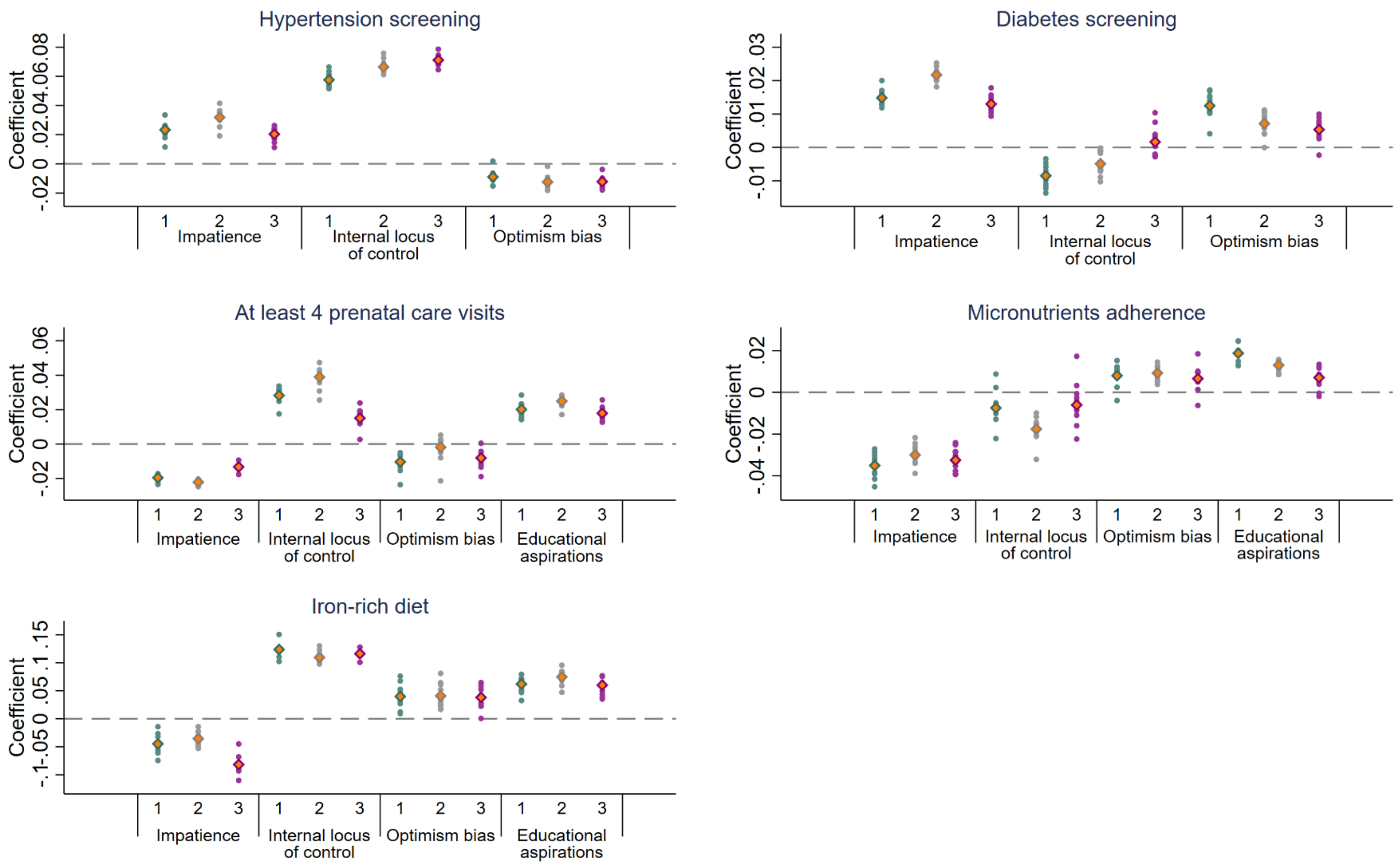

Notes: Each chart presents the coefficient of the behavioral predictors on our outcome of interest, which is presented on the title of each chart. The yellow diamond-shape presents the coefficient in a model with all 14 municipalities included. The dots represent the value of the coefficient of interest, excluding one of the 14 municipalities at a time. The green dots under over the value of 1 in the x-axis refer to our preferred specification which we include in Tables 6 and 7 in the document. The gray dots over the value of 2 in the x-axis, present the model without any controls. The purple dots over the value of 3 in the x-axis add facility fixed effects to the specification in addition to the individual and household controls. No facility controls or municipality fixed effects are included in this specification since they are collinear with the facility fixed effects. Section 4 in the paper describes the main specification and Table 1 includes the list of controls. The behavioral explanatory variables of interest are described as follows. Impatience is the standardized measure of the present bias index; internal locus of control is the standardized measure of the adapted locus of control index; optimism bias is the standardized measure of the general optimism bias Index; and educational aspirations is the standardized measure of the educational aspirations Index.

Appendix References

Anderson, M. (2008). Multiple Inference and Gender Differences in the Effects of Early Intervention: A Reevaluation of the Abecedarian, Perry Preschool, and Early Training Projects. Journal of the American Statistical Association. 103. 1481-1495.

Clarke, D. and Romano, J. and Wolf, M. (2020). The Romano-Wolf Multiple Hypothesis Correction in Stata. SSRN Electronic Journal.

Falk, A., Becker, A., Dohmen, T., Huffman, D., & Sunde, U. (2022). The preference survey module: A validated instrument for measuring risk, time, and social preferences. *Management Science*.

List, J., Shaikh, A. and Xu, Y. (2016). "Multiple Hypothesis Testing in Experimental Economics" Artefactual Field Experiments 00402, The Field Experiments Website.

Oster, Emily. (2016). Unobservable Selection and Coefficient Stability: Theory and Evidence. Journal of Business & Economic Statistics. 37.
