# Supplementary material for: Do behavioral drivers matter for healthcare decision-making during crises? A study of low-income women in El Salvador during the COVID-19 pandemic
Source: BMC Public Health. 2024 Aug 6;24:2122. doi: 10.1186/s12889-024-19039-y (PMC11302350; doi:10.1186/s12889-024-19039-y)
Supplement: Supplementary file 2 — Supplementary Material 2 [file 12889_2024_19039_MOESM2_ESM.pdf]

HOUSEHOLD SURVEY FOR HEALTH CARE UTILIZATION FOR WOMEN AND CHILDREN
EL SALVADOR 2022
ENGLISH TRANSLATION FROM SPANISH

Field	Question	Answer
municipality *(required)*	Select Ecos F Municipality	
unit *(required)*	Select Ecos F Name	
interviewer *(required)*	Select Interviewer	
num *(required)*	House number	
zone *(required)*	Select Housing Zone	
intro_label	Hello good morning/afternoon my name is [enumerator] (show ID) and I am working on a Salud Mesoamerica Initiative project for the Ministry of Health, on the health of women, mothers, children and girls throughout the country through a short survey. We would like to ask a few questions to the head of the household (or the household member who more knowledgeable about the home)?	
available *(required)*	Could you speak to the caretaker/head of household (or the household member who has more knowledge about the home)?	1 Yes<br>0 No
consent *(required)*	Did you obtain informed consent?	1 Yes<br>0 No

got_consent
Group re/evans chen. se/ec/ed{ S/consent/ , 'J g

Field	Question	Answer
filtro_menores5 *(required)*	Are there children under 5 years of age living in this dwelling?	1 Yes<br>0 No
filter_mef *(requiried)*	Does this household contain women between 15 and 49 years of age?	1 Yes<br>0 No

obtained consent > mef survey
Group re/evans rehen. se/ecfed{ S////lra meg . 'f g

Field	Question	Answer
members_note	Members of the household	

obtained consent > mef survey > Household Members: Sociodemographic information on household members

Field	Question	Answer
n_household *(required)*	How many people live in this dwelling counting babies and elderly people?	
hh_members	I am going to ask you some questions about the members of this household starting with the oldest and going down through the youngest in descending order. Fill in the information for	
	each member of the household and then move on to the next member. Be sure to register everyone the members of the household, and that the total number of items corresponds to the [n_household]persons mentioned above.	
obtuvo_consent > encuesta_mef > Members Household: Socio-demographic information of household members > Be sure to register all members of the household.<br>members of the household, and that the total number of rows corresponds to the [n_household] persons mentioned above. (1)		(Repeated group)
member_id *(required)*	Member ID number (assign a number for each member in the household):	
member_sex *(required)*	Is it male or female?	1 Male<br>2 Woman
miembro_edad_ano *(required)*	How old was your last birthday? If less than 1 year old, please enter 0<br>*Response constrained to: . " -- 0 and .< 105*	
member_age_month *(required)*	How old are you (months)?<br>For children under 5 years of age write the age in years and months.<br>*Question relevant when: selected(${year_old_member) , '1') or selected(${year_member} , '2') or selected($(year_member} , '3') or selected(${year_old_member} , '4)*<br>*Response constrained to: . >-- 0 and .< 60*	
mother_id_member *(required)*	if the mother resides in the household, enter the mother's identification (ID) number. If she does not reside in the household, enter 77<br>*Question relevant when: selected($(age_member) , '1') or selected(${year_old_member} , '2) or selected(${year_old_member} , '3') or selected($(age_member} , '4)*	
parent_id_member *(required)*	if the father resides in the household, enter the father's identification (ID) number. If he does not reside in the household, enter 77<br>*Question relevant when: selected($(age_member) , '1') or selected(${year_old_member} , '2) or selected(${year_old_member} , '3') or selected(${year_old_member} , '4)*	

informant *(required)*	Is this person the informant?<br>*Question relevant when: ${miembro_edad_ano) >=15*	1	Yes
		0	No

obtuvo_consent > encuesta_mef > hh_elegible

Group *relevant when.' ${hh_womanligible_1} --1 or ${hh_womanligible_2} --1 or ${hh_womanligible_3} --1 or $(hh_womanligible_4} --1 or $(hh_womanligible_5) --1 or ${hh_mu)ereligible_6} --1 or*

${hh womaneligible 7} --1 or ${hh womaneligible 8] --1 or ${hh womaneligible* 9/ =1 or *${hh womaneligible 10} --1 or ${hh womaneligible 11} --1 or ${hh womaneligible 1 2} --1 or

${hh womaneligible 13} --1 or ${hh womaneligible 14) --1 or ${hh womaneligible Y5} *=1*

housing_inro	**Housing Characteristics**<br><br>I will now ask you some questions about housing.		
n_rooms *(required)*	Excluding bathrooms, kitchen, hallway, and garage, how many rooms does the dwelling have?		
bedrooms *(required)*	How many rooms are used exclusively for sleeping quarters?		
material_techo *(required)*	Is the predominant roofing material...?	1	Asbestos/Duralite
		2	Metal (Sheet)
		3	Clay/cement tile
		88	Another
material_walls *(required)*	Is the predominant wall material...?	1	Brick, block, tile
		2	Adobe
		3	Bahareque
		8<br>8	Another
material_floor *(required)*	The predominant material of the floor is...?	1	Ceramic, brick or cement
		2	Earth
		88	Another
luz *(required)*	Does this home have electric light...?	1	Yes
		0	No
telephone *(required)*	Does this dwelling have a landline telephone...?	1	Yes
		0	No
water *(required)*	How do you treat the water you drink?	0	None
		1	Boil it
		2	Treated with bleach or puriagua
		3	Uses water filter
		4	Buy bottled water
		88	Other
latrine *(required)*	Do you have a latrine or toilet in this dwelling?	1	Yes

		0	No
type_letrine *(required)*	What type of latrine or toilet facilities do you have?<br>*Question relevant when: selected($(latrine} , '1)*	1	Washing toilet connected to sewer
		2	Toilet connected to septic tank
		3	Dry pit latrine
		4	Composting latrine
		5	Untreated flush toilet
		88	Other type of latrine
residuals *(required)*	How is gray (waste) water disposed of in the home?	1	Disposal is to sewer
		2	By sump well system
		3	Open sky to the site
		4	To the street
		5	Streams, rivers or other places
subsidies *(required)*	What benefits does the household receive from the government? Check all that apply	1	None
		2	Gas Subsidy
		3	Electric Energy Subsidy
		4	Solidarity Communities Bonds Rurales
		5	Solidarity Communities Bonds Urban
		6	Universal Basic Pension
		7	School package
		8	Agricultural package
		9	Demobilized Pension
		88	Another
subsidies_other	Other benefits:<br>*Question relevant when: selected(${subsidies} , '88')*		
covid_note	Effect of COVID-19 in the home<br><br>Now I am going to ask you a few questions about how the COVID-19 pandemic has affected your household.		
change_entry *(required)*	Since March 2020 to date, due to the COVID-19 pandemic, your household income has been...	1	Reduced
		2	Increased
		3	They have not changed
		98	Do not know
		99	No response
covid_revenue_reduc *(required)*	If household income decreased, for what reason did household income decrease? Check all that apply<br>*Question relevant when: selected($(input_change) , '1)*	1	I could not go to work
		2	Loss of employment of any household member
		3	Reduced working hours salary
		4	He would not be able to open his business
		5	Could not harvest or sow
		6	Illness or death of any family member
		88	Other (specify)
other_revenue_reduc *(required)*	Other reason (explain)<br>*Question relevant when: selected($(covid_revenue_reduc} , '88')*		
covid_diag_member *(required)*	Have you or any household member ever been diagnosed with COVID-19 by test or by healthcare personnel?	1	Yes
		0	No
		98	Do not know
		9 9	No response
oovid_diag_miembro_hosp *(required)*	Who was diagnosed with COVID-19, did they require hospitalization?<br>*Question relevant when: selected(${covid_diag_member} ,'1')*	1	Yes
		0	No
		98	Do not know
		99	No response
covid_other_service *(required)*	At any time since the start of the pandemic, i.e., March 2020 to date, did you or anyone in your	1	Yes
		0	No

	household need medical care or health services for conditions other than coronavirus, but did NOT receive it because of the coronavirus pandemic?	98 Do not know<br>99 No response
covid_another_service_eng *(required)*	What type of medical care did you need (open-ended question)?<br>*Question relevant when: selected(${covid_other_service} , '1')*	
covid_another_service_no *(required)*	Why did you or the household member who needed medical care or services not receive them? Check all that apply<br>*Question relevant when: selected($(covid_other_service} , '1')*	1 The health unit was closed<br>2 The health unit was not accepting patients at that time. moment<br>3 No appointments were available<br>4 I went to the unit, but I did not attended<br>5 I was afraid of contagion from the COVID-19<br>6 I preferred to wait for the COVID-19 infections<br>7 I had to take care of some household member<br>8 Difficulty with transportation to unity<br>9 I had symptoms of COVID-19 and had to stay at home<br>10 I had financial difficulties to pay for the service<br>88 Other (specify)
other_reason_no_serv *(required)*	Otra razón por la cual no recibió atención médica o servicios de salud<br>*Question relevant when: selected($(covid_other_service_no} , '88')*	
cevid_service_tele *(required)*	At any time since the start of the pandemic, i.e. March 2020 to date, have you or any member of your household had an appointment with a doctor, nurse or other health care professional by video or telephone?	1 Yes, by video<br>2 Yes, by phone<br>0 No<br>98 Do not know<br>99 No response
women_note	Women from 15 to 49 selected	
obtuvo_consent > encuesta_mef > hh_elegible > Add information for each woman aged 15-49 living in this household (1)		(Repeated group)
repeat_woman_note	For each woman aged 15-49, ask all of the following questions.<br><br>Add information for up to 2 women ages 15 to 49 living in this household.	
woman_informant	**Interviewer:** *Is this woman the same as the one who answered the housing section and day the consent to the survey?*	1 Yes<br>0 No
consent_woman /required}	Did you obtain informed consent?	1 Yes<br>0 No
	Interviewer: If you have already interviewed two women, and there are more than two in the home, select that you did not obtain consent for the rest of the women to end the survey. *Question relevant when: selected($(woman_informant) , '0')*	
obtained_consent > mef_survey > hh_elegible > Add information for each woman aged 15 to 49 living in this household (1) > obtained_consent2<br>*Group relevant when: Selected($(consent_woman} , '1') or selected($(consent_woman} , '1')*		
woman_car_note	Characteristics of Women	
got_consent > survey_mef > hh_elegible > Add information for each woman aged 15-49 living in this household (1) > got_consent2 > Now I would like to ask you some questions about yourself.		
female_age *(required)*	What is your age?<br>*Enter 98 if don't know, 99 if don't respond*	
status_civil_woman {required}	What is your marital status?	1 Single (never married)<br>2 Married<br>3 Accompanied/Free union<br>4 Divorced<br>5 Separated<br>6 Widowed<br>88 Another<br>98 Do not know<br>99 No response

medical_insurance *(required)*	Do you have any medical insurance?	1 It does not have<br>2 ISSS<br>3 Teachers' Welfare<br>4 IPSFA<br>5 Collective<br>6 Individual (private)<br>88 Another<br>98 Do not know<br>99 No response
leer_type *(required)*	Can you read and write?	1 Yes<br>0 No<br>98 Do not know<br>99 No response
estudia *(required)*	Are you currently studying?	1 Yes<br>0 No<br>98 Do not know<br>99 No response
note_nivel	What was the last level studied and grade you passed?	
educacion_nivel *(required)*	Education level	0 None<br>1 Initial education<br>2 Kindergarten<br>3 Elementary<br>4 High school<br>5 Higher-University<br>6 Higher-Non-University<br>7 Special Education<br>98 Do not know<br>99 No response
education *(required)*	Grade of education	
health *(required)*	Thinking about the last 30 days, would you say your health has been Very Good, Good, Fair, Bad or Very Bad?	1 Very good<br>2 Good<br>3 Regular<br>4 Bad<br>5 Very bad<br>98 Do not know<br>99 No response
woman_services_note	Use of women's health services	
reunido_promotor *(required)*	In the last 3 months, have you met with or been visited by a CHW in your home or community?	1 Yes<br>0 No<br>98 Do not know
		9 9 No response
reason_no_visit *(required)*	Why hasn't a health promoter met or visited you?<br>*Question relevant when: selected(${rejoined promotor) , '0')*	1 They have not come to visit me<br>2 I have not wanted to meet for fear to COVID-19<br>3 They have come to visit me, but not I have been able to serve them<br>4 I was not at home when came to visit<br>5 I have no interest in the services of the promoter<br>88 Other (specify).
another_reason_not_to_visit *(required)*	Another reason why you have not met with or been visited by a health promoter<br>*Question relevant when: selected($(reason_no_visit} , '88)*	

obtained consent > mel survey > hh eligible > Add information for each woman aged 15-49 living in this household (1) > obtained consent2 > promotor

Group relevant when.' selected(8(gathered promoter) , '1')

promoter_note	Did the community health promoter provide any of the following services to you in the last threemonths. ..?<br>Ask for each item and record the answer.	
promoter_label	*Service*	
cuidados_pre *(required)*	Referral for prenatal screening or enrollment	1 Yes<br>0 No

		98 Do not know<br>99 No response
referencia_parto *(required)*	Referral for delivery in an institution	1 Yes<br>0 No<br>98 Do not know<br>99 No response
care_post *(required)*	Referral for postnatal care	1 Yes<br>0 No<br>98 Do not know<br>99 No response
tips *(required)*	Family planning and contraceptive advice	1 Yes<br>0 No<br>98 Do not know<br>99 No response
childhood_vaccination (required)	Childhood Immunization	1 Yes<br>0 No<br>98 Do not know<br>99 No response
nutrition *(required)*	Child nutrition tips	1 Yes<br>0 No<br>98 Do not know<br>99 No response
information *(required)*	Information, education and communication sessions (educational talks or counseling)	1 Yes<br>0 No<br>98 Do not know<br>99 No response
home_visit *(required)*	In the last 3 months, have you met with or been visited by health personnel (doctors or nurses) atyour home to provide you with any services?	1 Yes<br>0 No<br>98 Do not know<br>99 No response
service_domicilum_visit *(required)*	What service were you provided on that visit?<br>*Question relevant when: selected($(home_visit} , '1')*	
visit_domicilumbecause *(required)*	Why has a doctor or nurse not met or visited you at your home?<br>*Question relevant when: selected(${home_visit} , '0')*	1 They have not come to visit me<br>2 I have not wanted to meet for fear<br>to COVID-19<br>3 They have come to visit me, but not<br>I have been able to serve them<br>4 I was not at home when<br>came to visit<br>5 I have no interest in the services<br>of the promoter<br>88 Other (specify).
other_visit_domicilum_reason *(required)*	Other, specify<br>*Question relevant when: selected(${visit_domicilumbecause} , '88')*	

obtuvo_consent > encuesta_mef > hh_elegible > Add information for each woman aged 15-49 living in this household (1) > obtuvo_consent2 > detection

detection_note	During the last 6 months has been performed in any health facility the...	
label_detection	Service	
diabetes *(required)*	Diabetes screening (sugar test)?	1 Yes<br>0 No<br>98 Do not know<br>99 No response
hypertension *(required)*	Hypertension screening (blood pressure measurement)?	1 Yes<br>0 No<br>98 Do not know<br>99 No response
cytology *(required)*	Cervical cancer screening (cytology or pap smear)?	1 Yes<br>0 No<br>98 Do not know<br>99 No response
covid_woman_note	**COVID-19 awareness and use of health services during the pandemic by women.**	
covid_avoid_attention *(required)*	At some point since the start of the pandemic, i.e., March 2020 to the present, you will<br>did you avoid or postpone medical care for any of the following services? Check all that apply.	1 Family Planning<br>2 Cytology or Pap smear

		3 Preconception care<br>4 Diabetes screening or control<br>5 Detection or control of hypertension/high blood pressure<br>88 Other (specify)<br>0 None of the above<br>98 Do not know<br>99 No response
other_covid_avoid_attention *(required)*	Other service avoided or postponed<br>*Question relevant when: selected(${covid_avoid_attention} , '88')*	
covid_avoid_attention_why *(required)*	Why did you avoid or postpone medical care for those services?<br>Check all that apply<br>*Question relevant when: selected(${covid_avoid_attention_avoidance} , '1') or selected($(covid_avoid_attention} , '2') or selected($(covid_avoid_attention} , '3') or selected($(covid_avoid_attention) , '4') or selected($(covid_avoid_attention) , '5') or selected($(covid_avoid_attention) , '88')*	1 The health unit was closed<br>2 The health unit was not acceptingpatients at that time. moment<br>3 No appointments were available<br>4 I went to the unit, but I did not attended<br>5 I was afraid of contagion from the COVID-19<br>6 I preferred to wait until the COVID-19 infections<br>7 I had to take care of some household member<br>8 Had difficulty in transportation to unity<br>9 I had symptoms of COVID-19 and had to stay at home<br>10 I had financial difficulties to pay for the service<br>88 Other (specify)
other_r_avoid_attention *(required)*	Other reason for avoiding or postponing medical care for those services<br>*Question relevant when: selected($(covid_avoid_attencion_why) , '88')*	
note_condition_salud	I would like to ask you now about your health condition.	
covid_risk_factors *(required)*	Do you have any of the following conditions? Check all that apply.	1 Hepatitis<br>2 Tuberculosis<br>3 Hypertension<br>4 Diabetes<br>5 Chronic kidney disease<br>6 Cancer<br>7 Cardiovascular disease<br>8 Asthma<br>9 Obstructive pulmonary disease<br>10 Immune system disease<br>11 None<br>88 Another chronic disease (specify)
other_risk_factor *(required)*	Other chronic disease (specify)<br>*Question relevant when: selected(${covid_risk_factors} , '88')*	
note knowledge_covid	Now I am going to ask you some questions about the coronavirus (COVID-19) and how you feel about some of the changes that have occurred as a result of the pandemic. There are no right or wrong answers.	
obtuvo_consent > encuesta_mef > hh_elegible > Add information for each woman aged 15 to 49 living in this household (1) > obtuvo_consent2 > covid_acciones		
covid_actions_note	In your opinion, how effective are the following actions in keeping you safe from COVID?	
oovid_labels_actions	*Actions*	
covid_mascarilla *(required)*	Use a mask	1 Not effective<br>2 Something effective<br>3 Very effective<br>98 Do not know
covid_lavarse *(required)*	Wash hands with soap or use hand sanitizer frequently.	1 Not effective<br>2 Something effective

		3	Very effective
		98	Do not know
oovid_avoid_spaces (required)	Avoid public spaces, meetings and crowds	1	Not effective
		2	Something effective
		3	Very effective
		98	Do not know
covid_avoid_hosp (required)	Avoid hospitals and clinics	1	Not effective
		2	Something effective
		3	Very effective
		98	Do not know
covid_avoid_transport (required)	Avoid public transportation	1	Not effective
		2	Something effective
		3	Very effective
		98	Do not know
obtuvo_consent > encuesta_mef " hh_elegible > Add information for each woman aged 15 to 49 living in this household (1) > obtuvo_consent2 > ultima_semana			
covid_last_week	In the past week, how regularly have you been doing the following? (Read options for answer)		
covid_7days_labels	*Activity*		
covid_7days_mask (required)	I wore a mask when I went anywhere away from home.	1	Always
		2	Sometimes
		3	Never
		4	Not applicable
		99	Refused to answer
covid_7days_distance (required)	I kept a distance of 1 meter from others in all public places.	1	Always
		2	Sometimes
		3	Never
		4	Not applicable
		99	Refused to answer
covid_7days_disinfect (required)	I disinfected my hands anywhere outside the home	1	Always
		2	Sometimes
		3	Never
		4	Not applicable
		99	Refused to answer
covid_7days_disinfect 2 (required)	I disinfected all surfaces and objects around me.	1	Always
		2	Sometimes
		3	Never
		4	Not applicable
		99	Refused to answer
covid_vaccinated (required)	Have you ever been vaccinated against COVID-19?	1	Always
		2	Sometimes
		3	Never
		4	Not applicable
		99	Refused to answer
covid_dosage (required)	Did you receive all the required doses?	1	Yes
		0	No
covid_vaccine_interest (required)	Once you have access to the COVID-19 vaccine, you will...	1	Would definitely get vaccinated
		2	Would probably get vaccinated
		3	Not sure if you would get vaccinated
		4	Probably would not vaccinate
		5	Definitely would not vaccinate
covid_reason_no_vaccine (required)	For which of the following reasons would you not get vaccinated? Check all that apply.	1	I am concerned about possible side effects of a COVID-19 vaccine.
		2	I don't know if a COVID-19 vaccine will work.
		3	I don't think I need a COVID-19 vaccine.
		4	I don't like vaccines
		5	My doctor has not recommended that I get the COVID-19 vaccine.
		6	I want to wait to see if it is safe and maybe get vaccinated later.
		7	I think other people need it more than I do right now.
		8	I am concerned about the cost of the COVID-19 vaccine.
		9	I don't trust the COVID-19 vaccine.
		10	I don't trust the government

			88	Other reason (please specify)
other_reason_no_vaccine_covid (required)	Another reason why you would not get vaccinated<br>*Question relevant when: selected(${covid_reason_no_vaccine} , '88)*			
covid_reason_no_vaccine_why (required)	Why do you think you do not need a COVID-19 vaccine? Check all that apply.		1	I already had COVID-19
			2	I am not part of a high-risk group
			3	I plan to use facemasks/mouth masks or other precautions instead of vaccination
			4	I do not believe that COVID-19 is a serious disease.
			5	I do not believe vaccines are beneficial
			88	Other reason (please specify)
other_reason_not_need_vaccine (required)	Another reason you think you don't need a COVID-19 vaccine<br>*Question relevant when: selected(${covid_reason_no_vaccine_why} , '88)*			

Field	Question	Answe
covid_media_information *(required)*	In the last week, which of the following means of information do you use to obtain information about COVID-19? Check all that apply.	1 Twitter<br>2 Facebook<br>3 Whatsapp<br>4 Newspaper<br>5 Internet<br>6 Television<br>7 Radio<br>8 Health personnel<br>9 Conversations with friends and family members
covid_media_info_prim *(required)*	Of the means you mentioned, what is the main one you use to obtain information from COVID?<br>19? Check only one.	1 Twitter<br>2 Facebook<br>3 Whatsapp<br>4 Newspape<br>5 rInternet<br>6 Television<br>7 Radio<br>8 Health personnel<br>9 Conversations with friends and family members

obtained consent > mel survey > hh eligible > Add information for each woman aged 15-49 living in this household (1) > obtained consent2 >

covid confia covid_confia_note<br>covid_confia_label	How much do you trust the following sources of information?<br>*Source of information*	1 Does not trust anything<br>2 Trust something<br>3 Trust a lot
covid_conf_friends *(required)*	Friends or relatives	1 Does not trust anything<br>2 Trust something<br>3 Trust a lot
covid_conf_doctor *(required)*	Doctors or other health personnel	1 Does not trust anything<br>2 Trust something<br>3 Trust a lot
covid_conf_gob_nac *(required)*	Government authorities	1 Does not trust anything<br>2 Trust something<br>3 Trust a lot
covid_conf_moh *(required)*	The Ministry of Health	1 Does not trust anything<br>2 Trust something<br>3 Trust a lot
covid_conf_oms *(required)*	The World Health Organization (WHO)	1 Does not trust anything<br>2 Trust something<br>3 Trust a lot
note_fertilty	**Fertility**	
pregnant_before *(required)*	Now I am going to ask you some questions about the pregnancies and children you have had. Have you ever been pregnant?	1 Yes<br>0 No<br>98 Do not know<br>99 No response

Group relevant when: selected(${embarazada_antes} , '1')

obtuvo_consent > encuesta_mef > hh_elegible > Add information for each woman aged 15-49 living in this household) > obtuvo_consent2 > Fertility and pregnancies

children *(required)*	How many sons and how many daughters did you have in total who were born alive?<br>Born alive means that the baby cried or showed some sign of life.<br>*Enter 98 if don't know, 99 if don't answer*<br>*Question relevant when: selected(${pregnant_before} , '1')*	
pregnant *(required)*	Are you currently pregnant?	1 Yes<br>0 No<br>98 Do not know<br>99 No response
wanted_pregnancy *(required)*	When you became pregnant did you want to become pregnant at that time?<br>*Question relevant when: selected(${pregnant} , '1')*	1 Yes<br>0 No<br>98 Do not know<br>99 No response

wanted_to_wait *(required)*	Did you want to wait to have a baby later or did she not want to have any more children?<br>*Question relevant when: selected(${pregnancy_wanted} , '0') or Selected(${wanted_pregnancy) , '98') or selected(${wanted_pregnancy] , '99')*	1 I wanted to wait<br>2 I didn't want to have any more children<br>98 Do not know<br>99 No response
obtuvo_consent > encuesta_mef > hh_elegible > Add information for each woman aged 15 to 49 living in this household (1) > obtuvo_consent2 > family planning		
pf_intro	**Family Planning**<br><br>I am going to ask you some questions about family planning, that is, how to plan the sons or daughters you want to have, with methods that a couple can use to delay or avoid pregnancy.	
obtained consent > mef survey > eligible hh > Add information for each woman aged 15-49 living in this household (1) > obtained consent2 > family planning > pl oido		
pf_oido_note	Have you heard of...	
pf_labels_hearing	*Method*	
pf_oido_diu *(required)*	IUD (Intra-Uterine Device): *A doctor or nurse can fit women with an IUD device. Sometimes referred to as a device or a copper T.*	1 Yes<br>0 No<br>98 Do not know<br>99 No response
pf_oido_inectable *(required)*	Injectable: *Women can receive an injection from health personnel that prevents them from becoming pregnant for a month or more.*	1 Yes<br>0 No<br>98 Do not know<br>99 No response
pf_hearing_implants *(required)*	Implant: *A doctor or nurse can place one or more sticks in a woman's arm, which can prevent pregnancy for 12 months or more.*	1 Yes<br>0 No<br>98 Do not know<br>99 No response
pf_oido ildora *(required)*	Pill: *Women can take a pill every day to prevent pregnancy.*	1 Yes<br>0 No<br>98 Do not know<br>99 No response
pf_oido_cond *(required)*	Condom: *Men can put on a rubber condom before having sex.*	1 Yes<br>0 No<br>98 Do not know<br>99 No response
pf_oido_cond_fem *(required)*	Female Condom: *Women can put a condom in the vagina before having sexual intercourse.*	1 Yes<br>0 No<br>98 Do not know<br>99 No response
pf_hearing_diaphragm *(required)*	Diaphragm: *This method consists of a flexible metal ring with a latex membrane, which is inserted into the vagina to prevent pregnancy.*	1 Yes<br>0 No<br>98 Do not know<br>99 No response
pf_oido_espon *(required)*	Spermicidal Sponge/Jelly: *This method consists of a jelly that is applied inside the vagina, with an applied to the vagina.*	1 Yes<br>0 No<br>98 Do not know<br>99 No response
pf_oido_anti_emer *(required)*	Emergency contraception: *As an emergency measure, within three days after unprotected intercourse, women can take special pills to prevent p r e g n a n c y , the morning-after pill or emergency contraception.*	1 Yes<br>0 No<br>98 Do not know<br>99 No response
pf_oido_est_fem *(required}*	Female sterilization: *women can undergo an operation to stop having children*	1 Yes<br>0 No<br>98 Do not know
		99 No response
pf oido_est mas *(required)*	Male sterilization: *men can have surgery to prevent them from having children. It is known as vasectomy.*	1 Yes<br>0 No<br>98 Do not know<br>99 No response
pf_oido_other_mod *(required)*	Another modern method	1 Yes<br>0 No<br>98 Do not know<br>99 No response
pf_oido_otro_trad *(required)*	Another traditional method	1 Yes<br>0 No

		98 Do not know<br>99 No response
method_type *(required)*	What method is that?<br>*Question relevant when: selected(${pf_oido_otro_mod) , '1') or selected($(pf_oido_otro_trad} ,'1)*	
obtained consent > mef survey > eligible hh > Add information for each woman aged 15-49 living in this household (1) > obtained consent2 > family planning > pf usa		
pf_usa_note	Are you currently using	
pf_usa_labels	*Method*	
pf_usa_diu *(required)*	IUD (Intra Uterine Device)<br>*Question relevant when: selected($(pf_oido_diu} , '1')*	1 Yes<br>0 No<br>98 Do not know<br>99 No response
pf_usa_inyectable *(required)*	Injectable<br>*Question relevant when: selected($(pf_oido_inyectable) , '1)*	1 Yes<br>0 No<br>98 Do not know<br>99 No response
pf_usa_implants *(required)*	Implants<br>*Question relevant when: selected(${{pf_oido implants} , 'l)*	1 Yes<br>0 No<br>98 Do not know<br>99 No response
pf_usa_pildora *(required)*	Pill<br>*Question relevant when: selected($(pf_oido_pildora} , '1)*	1 Yes<br>0 No<br>98 Do not know<br>99 No response
pf_usa_cond *(required)*	Condom<br>*Question relevant when: selected(${pf_oido_cond} , '1')*	1 Yes<br>0 No<br>98 Do not know<br>99 No response
pf_usa_cond_fem *(required)*	Female Condom<br>*Question relevant when: se1ected(${pf_oido_cond_fem} , 'l)*	1 Yes<br>0 No<br>98 Do not know<br>99 No response
pf_usa_diaphragm *(required)*	Diaphragm<br>*Question relevant when: selected(${{pf_oido_diaphragm} , '1')*	1 Yes<br>0 No<br>98 Do not know<br>99 No response
pf_usa_espon *(required)*	Sponge/ Spermicidal jelly<br>*Question relevant when: selected(${pf_oido_opon} , '1')*	1 Yes<br>0 No<br>98 Do not know<br>99 No response
pf_usa_anti_emer *(required)*	Emergency contraception<br>*Question relevant when: selected($(pf_oido_anti_emer} , '1')*	1 Yes<br>0 No<br>98 Do not know<br>99 No response
pf_usa_other_mod *(required)*	Another modern method<br>*Question relevant when: selected($(pf_oido_otro_mod) , '1')*	1 Yes<br>0 No<br>98 Do not know<br>99 No response
pf_usa_other_trad *(required)*	Another traditional method<br>*Question relevant when: se1ected(${pf_oido otro trad} , 'l)*	1 Yes<br>0 No<br>98 Do not know<br>99 No response
obtained consent > mef survey > eligible hh > Add information for each woman aged 15-49 living in this household) > obtained consent2 > family planning > fp where		
pf_where_note	Where did you last get the...?	
pf_where_diu *(required)*	IUD (Intra Uterine Device)<br>*Question relevant when: selected(${pf_usa diu} , '1')*	1 Minsal establishment<br>2 Establishment of ISSS<br>3 Women's City<br>4 Pro-Family Clinic (ADS)<br>5 Clinic or private physician<br>6 ISBM<br>7 IPSFA<br>8 Pharmacy

		9	Minsal Promoter
		10	NGO promoter
		88	Another
		98	Do not know
		99	No response
pf_where_injectedble *(required)*	Injectable<br>*Question relevant when: selected(${pf_usa_inyectable) , '1)*	1	Minsal establishment
		2	Establishment of ISSS
		3	Women's City
		4	Pro-Family Clinic (ADS)
		5	Clinic or private physician
		6	ISBM
		7	IPSFA
		8	Pharmacy
		9	Minsal Promoter
		10	NGO promoter
		88	Another
		98	Do not know
		99	No response
pf_where_implants *(required)*	Implants<br>*Question relevant when: selected($(pf_usa_implantes} , 'l)*	1	Minsal establishment
		2	Establishment of ISSS
		3	Women's City
		4	Pro-Family Clinic (ADS)
		5	Clinic or private physician
		6	ISBM
		7	IPSFA
		8	Pharmacy
		9	Minsal Promoter
		10	NGO promoter
		88	Another
		98	Do not know
		99	No response
pf_where_pildora *(required)*	Pill<br>*Question relevant when: selected(${pf_usa_pildora} , '1')*	1	Minsal establishment
		2	Establishment of ISSS
		3	Women's City
		4	Pro-Family Clinic (ADS)
		5	Clinic or private physician
		6	ISBM
		7	IPSFA
		8	Pharmacy
		9	Minsal Promoter
		10	NGO promoter
		88	Another
		98	Do not know
		99	No response
pf_where_cond *(required)*	Condom	1	Minsal establishment
**Field**	**Question**	**Answer**	
	Question relevant when: se1ected(${pf_usa_cond} , '1')	2	Establishment of ISSS
		3	Women's City
		4	Pro-Family Clinic (ADS)
		5	Clinic or private physician
		6	ISBM
		7	IPSFA
		8	Pharmacy
		9	Minsal Promoter
		10	NGO promoter
		88	Another
		98	Do not know
		99	No response
pf_where_to_cond_fem {required}	Female Condom	1	Minsal establishment

	Question relevant when: selected(${pf_usa cond lem} , '1')	2	Establishment of ISSS
		3	Women's City
		4	Pro-Family Clinic (ADS)
		5	Clinic or private physician
		6	ISBM
		7	IPSFA
		8	Pharmacy
		9	Minsal Promoter
		10	NGO promoter
		88	Another
		98	Do not know
		99	No response
pf_where_diaphragm *(required)*	Diaphragm<br>*Question relevant when: selected($(pf_usa_diaphragm} , '1')*	1	Minsal establishment
		2	Establishment of ISSS
		3	Women's City
		4	Pro-Family Clinic (ADS)
		5	Clinic or private physician
		6	ISBM
		7	IPSFA
		8	Pharmacy
		9	Minsal Promoter
		10	NGO promoter
		88	Another
		98	Do not know
		99	No response
pf_where_is_espon *(required)*	Sponge/ Spermicidal jelly<br>*Question relevant when: selected(${pf_usa_espon} , '1')*	1	Minsal establishment
		2	Establishment of ISSS
		3	Women's City
		4	Pro-Family Clinic (ADS)
		5	Clinic or private physician
		6	ISBM
		7	IPSFA
		8	Pharmacy
		9	Minsal Promoter
		10	NGO promoter
		88	Another
		98	Do not know
		99	No response
pf_where_anti_emer *(required)*	Emergency contraception<br>*Question relevant when: selected(${pf_usa_anti_emer} , '1')*	1	Minsal establishment
		2	Establishment of ISSS
		3	Women's City
		4	Pro-Family Clinic (ADS)
		5	Clinic or private physician
		6	ISBM
		7	IPSFA
		8	Pharmacy
		9	Minsal Promoter
		10	NGO promoter
		88	Another
		98	Do not know
		9 9	No response
pf_where_another_mod *(required)*	Another modern method<br>*Question relevant when: selected($(pf_usa_otro_mod) , '1')*	1	Minsal establishment
		2	Establishment of ISSS
		3	Women's City
		4	Pro-Family Clinic (ADS)
		5	Clinic or private physician
		6	ISBM
		7	IPSFA
		8	Pharmacy

		9 Minsal Promoter<br>10 NGO promoter<br>88 Another<br>98 Do not know<br>9 9 No response
pf_where_another_trad *(required)*	Another traditional method<br>*Question relevant when: selected($(pf_usa_other_trad} , '1')*	1 Minsal establishment<br>2 Establishment of ISSS<br>3 Women's City<br>4 Pro-Family Clinic (ADS)<br>5 Clinic or private physician<br>6 ISBM<br>7 IPSFA<br>8 Pharmacy<br>9 Minsal Promoter<br>10 NGO promoter<br>88 Another<br>98 Do not know<br>99 No response

obtuvo_consent > encuesta_mef > hh_elegible > Aggregate information for each woman aged 15-49 living in this household (1) > obtuvo_consent2 > family planning > pf_como

pf_as_note	How did you last get the...?	
pf_as_diu *(required)*	IUD (Intra-Uterine Device)<br>*Question relevant when: selected($(pf_usa_diu) , '1')*	1 In the consultation in the unit of health, no appointment necessary<br>2 In the consultation in the unit of health, by making an appointment before<br>3 It was delivered to my home address<br>4 I attended a brigade of attention in my community<br>88 Other (specify)
pf_as_injectedble *(required)*	Injectable<br>*Question relevant when: selected(${{pf_usa_inyectable) , '1')*	1 In the consultation in the unit of health, no appointment necessary<br>2 In the consultation in the unit of health, by making an appointmentbefore<br>3 It was delivered to my home address<br>4 I attended a brigade of attention in my community<br>88 Other (specify)
pf_as_implants *(required)*	Implants<br>*Question relevant when: selected($(pf_usa_implants) , '1')*	1 In the consultation in the unit of health, no appointment necessary<br>2 In the consultation in the unit of health, by making an appointmentbefore<br>3 It was delivered to my home address<br>4 I attended a brigade of attention in my community<br>88 Other (specify)
pf_comopildora *(required)*	Pill<br>*Question relevant when: selected(${pf_usa_pildora} , '1')*	1 In the consultation in the unit of health, no appointment necessary<br>2 In the consultation in the unit of health, by making an appointmentbefore<br>3 It was delivered to my homeaddress
		4 I attended a brigade of attention in my community<br>88 Other (specify)
pf_as_cond *(required)*	Condom<br>*Question relevant when: selected(${pf_usa_cond} , '1')*	1 In the consultation in the unit of health, no appointment necessary<br>2 In the consultation in the unit of health, by making an appointmentbefore<br>3 It was delivered to my homeaddress

		4	I attended a brigade of attention in my community
		88	Other (specify)
pf_as_cond_fem *(required)*	Female Condom<br>*Question relevant when: selected($(pf_usa_cond_fem} , '1')*	1	In the consultation in the unit of health, no appointment necessary
		2	In the consultation in the unit of health, by making an appointmentbefore
		3	It was delivered to my homeaddress
		4	I attended a brigade of attention in my community
		88	Other (specify)
pf_as_diaphragm *(required)*	Diaphragm<br>*Question relevant when: selected(${pf_usa_diaphragm} , '1')*	1	In the consultation in the unit of health, no appointment necessary
		2	In the consultation in the unit of health, by making an appointment before
		3	It was delivered to my homeaddress
		4	I attended a brigade of attention in my community
		88	Other (specify)
pf_aso_espon *(required)*	Sponge/ Spermicidal jelly<br>*Question relevant when: selected(${pf_usa espon} , '1)*	1	In the consultation in the unit of health, no appointment necessary
		2	In the consultation in the unit of health, by making an appointmentbefore
		3	It was delivered to my homeaddress
		4	I attended a brigade of attention in my community
		88	Other (specify)
pf_as_anti_emer *(required)*	Emergency contraception<br>*Question relevant when: selected($(pf_usa_anti_emer} , '1')*	1	In the consultation in the unit of health, no appointment necessary
		2	In the consultation in the unit of health, by making an appointmentbefore
		3	It was delivered to my homeaddress
		4	I attended a brigade of attention in my community
		88	Other (specify)
pf_as_another_mod *(required)*	Another modern method<br>*Question relevant when: selected(${pf_usa_otro_mod] , '1')*	1	In the consultation in the unit of health, no appointment necessary
		2	In the consultation in the unit of health, by making an appointmentbefore
		3	It was delivered to my homeaddress
		4	I attended a brigade of attention in my community
		88	Other (specify)
pf_as_another_trad *(required)*	Otro método tradicional<br>*Question relevant when: selected($(pf_usa_other_trad} , '1)*	1	In the consultation in the unit of health, no appointment necessary
		2	In the consultation in the unit of health, by making an appointmentbefore
		3	It was delivered to my homeaddress
		4	I attended a brigade of attention in my community
		88	Other (specify)
sterilization *(required)*	Have you undergone Female Sterilization?	1	Yes
		0	No
		98	Do not know
		99	No response
sterilization_couple *(required)*	Has your partner undergone Male Sterilization?	1	Yes

		0	No
		98	Do not know
		99	No response
where_est *(required)*	Where did you have the sterilization performed? Ques/ion *relevant when: selected($(sterilization} , 'l) or selected($(sterilization_couple } , '1')*	1	Minsal establishment
		2	Establishment of ISSS
		3	Women's City
		4	Pro-Family Clinic (ADS)
		5	Clinic or private physician
		6	ISBM
		7	IPSFA
		8	Pharmacy
		9	Minsal Promoter
		10	NGO promoter
		88	Another
		98	Do not know
		99	No response

obtuvo_consent > encuesta_mef > hh_elegible > Add information for each woman aged 15-49 living in this household (1) > obtuvo_consent2 > family planning > pf_info

pf_info_note	In the last 6 months, have any health promoters, nurses, or other health promoters or doctor provided you with information about.		
pf_info_label	*Method*		
pf_info_diu *(required)*	IUD (Intra Uterine Device)	1	Yes
		0	No
		98	Do not know
		99	No response
pf_info_inectable *(required)*	Injectable	1	Yes
		0	No
		98	Do not know
		99	No response
pf_info_implants *(required)*	Implants	1	Yes
		0	No
		98	Do not know
		99	NO response
pf_info_pildora (required)	Pildora	1	Yes
		0	No
		98	Do not know
		99	No response
pf_info_cond *(required)*	Condom	1	Yes
		0	No
		98	Do not know
		99	No response
pf_info_cond_fem *(required)*	Female Condom	1	Yes
		0	No
		98	Do not know
		99	No response
pf_info_diaphragm *(required)*	Diaphragm	1	Yes
		0	No
		98	Do not know
		99	No response
pf_info_espon *(required)*	Sponge/ Spermicidal jelly	1	Yes
		0	No
		98	Do not know
		99	No response
pf_info_anti_emer *(required)*	Emergency contraception	1	Yes
		0	No
		98	Do not know
		9 9	No response
pf_info_est_fem *(required)*	Female Sterilization	1	Yes
		0	No
		98	Do not know
		99	No response

pf_info_est_mas (required)	Male Sterilization	1 Yes<br>0 No<br>98 Do not know<br>99 No response
usa_metodo (required)	Interviewer: According to the previous questions, does the respondent use any of the following contraceptive methods?	1 Yes<br>0 No
reason (required)	Could you tell me why you are not using a method to avoid pregnancy? Check all that apply<br>*Question relevant when: selected(${usa_metodo} , '0)*	1 Does not have sexual relations(without sex life)<br>2 Never had relations sexual (Virgin)<br>3 Cannot become pregnant<br>4 Menopausal<br>5 Hysterectomy/ operated on the matrix<br>6 Missed menstrual period since last delivery<br>7 She was pregnant<br>8 The partner or a family member is opposes<br>9 Fear of side effects (or secondary) or that it hurts you<br>10 It had side effects (it made him damage)<br>11 Religious reasons<br>12 Wants to be pregnant<br>88 Another reason<br>98 Do not know<br>99 No response
reason_other (required)	Other reason (explain)<br>*Question relevant when: se1ected(${reason} , '88')*	
covid_plan_fam_no (required)	At any time since the beginning of the pandemic, i.e., March 2020 to date, did you need familyplanning services but did not receive them?	1 Yes<br>2 No<br>3 I don't need them
covid_plan_am_no_because (required)	Why did you not receive family planning services?<br>*Question relevant when: selected(${covid_plan_fam_no} , '1')*	1 The health unit was closed<br>2 The health unit was not accepting patients at that time. moment<br>3 No appointments were available<br>4 I went to the unit, but I did not attended<br>5 No methods were available<br>6 I was afraid of contagion from the COVID-19<br>7 I preferred to wait for the COVID-19 infections<br>8 I had to take care of some household member<br>9 Had difficulty in transportation to unity<br>10 I had symptoms of COVID-19 and had to stay at home<br>11 I had financial difficulties to pay for the service<br>88 Other (specify)
otra_r_no_serv_planifica (required)	Another reason why you did not receive family planning services<br>*Question relevant when: selected(${covid_plan_fam_plan_no_porgue} , '88)*	
pf_comments (required)	Comments on family planning section	

preferences_note	Preferences and aspirations<br><br>READ: The following statements refer to attitudes toward life and the future. Please respond to whatextent you agree with each of them on a scale of 1 to 5, where 1 is completely disagree and 5 is completely agree.		
locus_1 *(required)*	It is entirely up to you what happens in your life.	1	Completely disagree
		2	Somewhat at odds
		3	Neither agree nor disagree
		4	Somewhat in agreement
		5	Completely agree
		98	Do not know
locus_2 *(required)*	Compared to the others, you have achieved what you deserve.	1	Completely disagree
		2	Somewhat at odds
		3	Neither agree nor disagree
		4	Somewhat in agreement
		5	Completely agree
		98	Do not know
locus_3 *(required)*	What he achieves in his life depends, first and foremost, on fate or luck.	1	Completely disagree
		2	Somewhat at odds
		3	Neither agree nor disagree
		4	Somewhat in agreement
		5	Completely agree
		98	Do not know
locus_4 *(required)*	Most of the time others make decisions about your life.	1	Completely disagree
		2	Somewhat at odds
		3	Neither agree nor disagree
		4	Somewhat in agreement
		5	Completely agree
		98	Do not know
locus_5 *(required)*	Success comes from hard work.	1	Completely disagree
		2	Somewhat at odds
		3	Neither agree nor disagree
		4	Somewhat in agreement
		5	Completely agree
		98	Do not know
locus_6 *(required)*	When he encounters difficulties in his life, most of the time he doubts his abilities.	1	Completely disagree
		2	Somewhat at odds
		3	Neither agree nor disagree
		4	Somewhat in agreement
		5	Completely agree
		98	Do not know
locus_7 *(required)*	The opportunities you have in your life depend on the resources you have.	1	Completely disagree
		2	Somewhat at odds
		3	Neither agree nor disagree
		4	Somewhat in agreement
		5	Completely agree
		98	Do not know
locus_8 *(required)*	Your effort is more important than the skills you possess	1	Completely disagree
		2	Somewhat at odds
		3	Neither agree nor disagree
		4	Somewhat in agreement
locus_9 *(required)*	You have little control over the things that happen in your life	1	Completely disagree
		2	Somewhat at odds
		3	Neither agree nor disagree
		4	Somewhat in agreement

preferences2_note	READ: Now I am going to ask you some questions about your present and future preferences in a hypothetical situation.<br><br>Suppose you are given a choice between receiving a payment today or a payment 12 months from now. I am now going to present you with five scenarios. For each of these scenarios we would liketo know which one you prefer.	
impatience_0 (required)	Would you rather receive $10 today or $15.4 in 12 months?	1 Today<br>2 Within 12 months
impatience_1 (required)	Would you prefer to receive 10 dollars today or 12.5 dollars 12 months from now?<br>*Ques/ion relevant when: selected(${impatience_0} , '2)*	1 Today<br>2 Within 12 months
impatience_2 (required)	Would you rather receive $10 today or $11.2 12 months from now2?<br>*Question relevant when: selected(${impatience_1) , '2')*	1 Today<br>2 Within 12 months
impatience_3 (required)	Would you rather receive $10 today or $10.6 in 12 months?<br>*Question relevant when: selected($(impatience_2) , '2')*	1 Today<br>2 Within 12 months
impatience_4 (required)	Would you rather receive $10 today or $10.3 in 12 months?<br>*Question relevant when: selected(${impatience_3) , '2')*	1 Today<br>2 Within 12 months
impatience_5 (required)	Would you prefer to receive 10 dollars today or 10.9 dollars 12 months from now?<br>*Ques/ion relevant when: selected(${impatience_3) , '1)*	1 Today<br>2 Within 12 months
impatience_6 (required)	Would you prefer to receive 10 dollars today or 11.9 dollars 12 months from now2?<br>*Question relevant when: selected(${impatience_2) , '1')*	1 Today<br>2 Within 12 months
impatience_7 (required)	Would you rather receive $10 today or $12.2 in 12 months?<br>*Question relevant when: selected($(impatience_6) , '1')*	1 Today<br>2 Within 12 months
impatience_8 (required)	Would you rather receive $10 today or $11.6 in 12 months?<br>*Question relevant when: selected(${impatience_6) , '2'}*	1 Today<br>2 Within 12 months
impatience_9 (required)	Would you prefer to receive 10 dollars today or 13.9 dollars 12 months from now?<br>*Question relevant when: selected(${impatience 1) , '1)*	1 Today<br>2 Within 12 months
impatience_10 (required)	Would you rather receive $10 today or $13.2 in 12 months?<br>*Question relevant when: selected($(impatience_9) , '2')*	1 Today<br>2 Within 12 months
impatience_11 (required)	Would you rather receive $10 today or $12.9 in 12 months?<br>*Question relevant when: selected($(impatience_10) , '2)*	1 Today<br>2 Within 12 months
impatience_12 (required)	Would you rather receive $10 today or $13.6 in 12 months?<br>*Question relevant when: selected(${impatience_10) , '1)*	1 Today<br>2 Within 12 months
impatience_13 (required)	Would you prefer to receive 10 dollars today or 14.6 dollars 12 months from now?<br>*Question relevant when: selected(${impatience_9} , '1)*	1 Today<br>2 Within 12 months
impatience_14 (required)	Would you rather receive $10 today or $14.3 in 12 months?<br>*Question relevant when: selected($(impatience_13) , '2')*	1 Today<br>2 Within 12 months
impatience_15 (required)	Would you rather receive $10 today or $15 in 12 months?<br>*Question relevant when: selected($(impatience_13) , '1)*	1 Today<br>2 Within 12 months
impatience_16 (required)	Would you rather receive $10 today or $18.5 in 12 months?<br>*Question relevant when: selected(${impatience_0) , '1)*	1 Today<br>2 Within 12 months
impatience_17 (required)	Would you rather receive $10 today or $20.2 in 12 months' time?<br>*Question relevant when: selected($(impatience_16) , '1)*	1 Today<br>2 Within 12 months
impatience_18 (required)	Would you rather receive $10 today or $19.3 in 12 months?<br>*Question relevant when: selected($(impatience_17) , '2')*	1 Today<br>2 Within 12 months
impatience_19 (required)	Would you rather receive $10 today or $19.7 in 12 months?<br>*Question relevant when: selected($(impatience_18) , '1)*	1 Today<br>2 Within 12 months
impatience_20 (required)	Would you rather receive $10 today or $18.9 in 12 months?<br>*Question relevant when: selected($(impatience_18) , '2)*	1 Today<br>2 Within 12 months
impatience_21 (required)	Would you rather receive $10 today or $21.0 in 12 months?<br>*Question relevant when: selected(${impatience_17} , '1)*	1 Today<br>2 Within 12 months
impatience_22 (required)	Would you rather receive $10 today or $21.5 in 12 months?<br>*Question relevant when: selected(${impatience_21} , '1)*	1 Today<br>2 Within 12 months
impatience_23 (required)	Would you rather receive $10 today or $20.6 in 12 months?<br>*Question relevant when: selected($(impatience_21) , '2)*	1 Today<br>2 Within 12 months
impatience_24 (required)	Would you rather receive $10 today or $16.9 in 12 months?<br>*Question relevant when: selected($(impatience_16) , '2)*	1 Today<br>2 Within 12 months
impatience_25 (required)	Would you prefer to receive $10 today or $16.1 in 12 months?<br>*Question relevant when: selected($(impatience_24) , '2')*	1 Today<br>2 Within 12 months
impatience_26 (required)	Would you rather receive $10 today or $15.8 in 12 months?<br>*Question relevant when: selected($(impatience_25) , '2)*	1 Today<br>2 Within 12 months

impatience_27 *(required)*	Would you rather receive $10 today or $16.5 in 12 months?<br>*Question relevant when: selected($(impatience_25) , '1)*	1 Today<br>2 Within 12 months
impatience_28 *(required)*	Would you rather receive $10 today or $17.7 in 12 months' time?<br>*Question relevant when: selected{ $/impatience_24/ , '1)*	1 Today<br>2 Within 12 months
impatience_29 *(required)*	Would you rather receive $10 today or $17.3 in 12 months?<br>*Question irrelevant when: selected($(impatience_28) , '2)*	1 Today<br>2 Within 12 months
impatience_30 *(required)*	Would you rather receive $10 today or $18.1 in 12 months?<br>*Question relevant when: selected($(impatience_28) , '1)*	1 Today<br>2 Within 12 months

obtained consent > mef survey > eligible hh > Added information for each woman aged 15 to 49 living in this household (1) > obtained consent2 > trust

confidence_note	Below I am going to list events one by one. For each one, I am going to ask you to answer how likely you think it is that this event will happen to you in the future, COMPARED TO OTHER WOMEN OFA SIMILAR AGE TO YOURS IN YOUR COMMUNITY.<br><br>The scale goes from 0 to 10, where 0 is not at all likely and 10 is very likely. If you consider it equally likely, mark 5.	
overconfidence_2 *(required)*	Living more than 76 years	0 Not likely<br>1 1<br>2 2<br>3 3<br>4 4<br>5 Just as likely<br>6 6<br>7 7<br>8 8<br>9 9<br>10 Very likely<br>98 Do not know
overconfidence_3 *(required)*	To steal from me or someone in my family.	0 Not likely<br>1 1<br>2 2<br>3 3<br>4 4<br>5 Just as likely<br>6 6<br>7 7<br>8 8<br>9 9<br>10 Very likely<br>98 Do not know
overconfidence_4 *(required)*	That next year my family will have more income	0 Not likely<br>1 1<br>2 2<br>3 3<br>4 4<br>5 Just as likely<br>6 6<br>7 7<br>8 8<br>9 9<br>10 Very likely<br>98 Do not know
overconfidence_5 *(required)*	That my children grow up healthy and strong	0 Not likely<br>1 1<br>2 2<br>3 3<br>4 4<br>5 Just as likely<br>6 6<br>7 7<br>8 8

		9 9<br>10 Very likely<br>98 Do not know
overconfidence_6 *(required)*	That in the next few months I get sick with something.	0 Not likely<br>1 1<br>2 2<br>3 3<br>4 4<br>5 Just as likely<br>6 6<br>7 7<br>8 8<br>9 9<br>10 Very likely<br>98 Do not know
note_minors	Children under 5 years old	
minors5_has *(required)*	Do you have any children under the age of 5 living in the home (they can be biological children, children of children under the age of 5)?<br>foster or adoptive)?	1 Yes<br>0 No
n_children5 *(required)*	How many children under the age of 5 do you have living in the household?<br>*Question relevant when: selected($(minor5_haves/ , '1')*	

obtuvo_consent > encuesta_mef > hh_elegible > Add information for each woman aged 15 to 49 living in this dwelling) > obtuvo_consent2 > Minors 5 General

Group relevant when. selected($(child5 has} , '1')

minors5_intro	Under 5 years old general<br><br>The following questions refer to your children under five in general.	
note_diarrhea	Diarrhea treatment knowledge and practices	
diarrhea_treatment *(required)*	If one of your children under the age of 5 got sick with diarrhea, what treatments would you give him/her?<br><br>*DO NOT READ ANY OPTIONS. Check all the ones I mentioned.*	serum Oral serum<br>antibiotic Antibiotic<br>antimotility Antimotility agent (iodochlorine, enteroguanil, intestinomycin, pepto bismol)<br>zinc Zinc<br>home Home remedy/medication herbal<br>other_treatment Other treatment

obtained consent > mef survey > eligible hh > Add information for each woman 15 to 49 years old living in this household (1) > obtained consent2 > Under 5 General > diarrhea drinks

diarrhea_drinks_note	If your child under the age of 5 got sick with diarrhea, would you give him/her any of the following beverages2 (Read options).	
diarrhea_drinks_label	*Beverages*	1 Yes<br>0 No<br>98 Do not know<br>99 No response
sales *(required)*	A liquid made from a special packet known as oral rehydration salts (show image).	1 Yes<br>0 No<br>98 Do not know<br>99 No response
serum2 *(required)*	A prepackaged ORS liquid (packaged oral serum)?	1 Yes<br>0 No<br>98 Do not know<br>99 No response
fluido_casero *(required)*	A homemade liquid or fluid recommended by health personnel?	1 Yes<br>0 No<br>98 Do not know<br>99 No response

obtained consent > mef survey > eligible hh > Add information for each woman 15-49 years old living in this household (1) > obtained consent2 > Under 5 General > diarrhea med.

diarrhea_med_another	What else would you give your child under 5 years of age if he/she gets diarrhea? (Read options).	
diarrhea_med_label	*Treatment*	1 Yes<br>0 No

		98 Do not know<br>99 No response
a_antibiotic *(required)*	Antibiotic	1 Yes<br>0 No<br>98 Do not know<br>99 No response
d_antimotilty *(required)*	Antimotility agent (iodochlorine, enteroguanil, intestinomycin, pepto bismol)	1 Yes<br>0 No<br>98 Do not know<br>99 No response
d_zinc *(required)*	Zinc (Show image)	1 Yes<br>0 No<br>98 Do not know<br>99 No response
d_casero *(required)*	Home remedy/herbal medicine	1 Yes<br>0 No<br>98 Do not know<br>99 No response
d_other_treatment *(required)*	Other treatment	1 Yes<br>0 No<br>98 Do not know<br>99 No response
recibio_sales *(required)*	In the last 6 months, did you receive oral rehydration salts sachets? Show image	1 Yes<br>0 No<br>98 Do not know<br>99 No response
has_sales *(required)*	Do you have oral rehydration salts at home? Show image	1 Yes<br>0 No<br>98 Do not know<br>99 No response
obtained consent > mef survey > eligible hh > Add information for each woman aged 15-49 living in this household (1) > obtained consent2 > Under 5 General > anemia		
perception_prev_anemia_note	Perceived prevalence of anemia and nutritional deficiencies.<br><br>Now I am going to ask you some questions about anemia and nutrition in your children and in your community.	
overconfidence_1 *(required)*	Of every 10 children under 5 years old living in your community, how many do you think are anemic?<br><br>If you consider that none, score 0, if you consider that all score 10, if you say half of the children, select 5. If you do not know what anemia is, select that option.	0 None<br>1 1<br>2 2<br>3 3<br>4 4<br>5 Half of the<br>6 6<br>7 7<br>8 8<br>9 9<br>10 All<br>96 Does not know what anemia is<br>98 Do not know
overconfidence_7 *(required)*	Out of every 10 children under the age of 5 living in your community, how many do you think do NOT consume enough food to grow up healthy and strong?<br><br>If you consider none, score 0, if you consider all score 10, if you say half of the children, select 5. If you do not know what anemia is, select that option.	0 None<br>1 1<br>2 2<br>3 3<br>4 4<br>5 Half of the<br>6 6<br>7 7<br>8 8<br>9 9<br>10 All<br>96 Does not know what anemia is

			98	Do not know

	**Information on the youngest child**<br><br>Now I am going to ask you some questions about your youngest son.	
obtuvo_consent > encuesta_mef > hh_elegible > Add information for each woman aged 15 to 49 living in this dwelling (1) > obtuvo_consent2 > Minors S General > Under 5 years old (1)		(Repeated group)
child_sex *(required)*	Is it male or female?	1 Male<br>2 Woman
date_knows *(required)*	Do you know the date of birth of your minor child?	1 Complete date<br>2 Month and year only<br>98 Do not know<br>99 No response
date_nino	Date of birth DD-MM-YYYYY<br>*Question relevant when: selected($(date_knows) , '1')*	
nino_edad_ano *(required)*	How old was [child's name] on his or her last birthday?<br><br>*At home that is less than 1 year old, enter 0*<br>*Response constrained to: . " -- 0 and .< 6*	
aspirations_1 *(required)*	What is the highest level of complete education you would like your child [child's name] to achieve?	1 Incomplete elementary school<br>2 Complete elementary school<br>3 Incomplete middle school<br>4 High school complete<br>5 Higher incomplete<br>6 Higher complete<br>88 Don't know<br>99 No response
aspirations_2 *(required)*	What is the highest level of complete education that you believe your [child's name] child will attain?	1 Incomplete elementary school<br>2 Complete elementary school<br>3 Incomplete middle school<br>4 High school complete<br>5 Higher incomplete<br>6 Higher complete<br>98 Do not know<br>99 No response
aspirations_3 *(required))*	Imagine your child [child's name] at the age of 25. what occupation would you like him/her to have? Write down the answer.	
aspirations_4 *(required)*	Imagine your child [child's name] at the age of 25. what occupation do you think he/she will have? Write down the answer	
vaccines_recall	Vaccination according to the mother	
vaccines *(required)*	Did [child's name] ever receive a vaccination to prevent getting a disease, including vaccinations received in a national vaccination campaign?	1 Yes<br>0 No<br>98 Do not know<br>99 No response
vaccinations_note	Tell me if [child's name] has received any of the following vaccinations<br>*Question relevant when: selected(${vaccines) , '1')*	
measles *(required)*	A measles, rubella and mumps (MMR) vaccine or MMR shot, which is a shot in the arm at 12 months of age or older.<br>*Question relevant when: selected(${vaccines} , '1')*	1 Yes<br>0 No<br>98 Do not know<br>99 No response
sarampion_c *(required)*	How many doses did you receive?<br>*Question relevant when: selected(${sarampion} , '1')*	1 1 Dose<br>2 2 doses<br>3 More than 2 doses<br>98 Do not know<br>99 No response
bcg *(required)*	BCG vaccine against tuberculosis, which is an injection in the arm or shoulders that usually leaves a scar.<br>*Question relevant when: selected(${vaccines} , '1')*	1 Yes<br>0 No<br>98 Do not know<br>99 No response
bcg_c *(required)*	How many doses did you receive?<br>*Question relevant when: selected(${bcg} , '1')*	1 1 Dose<br>2 2 doses<br>3 More than 2 doses<br>98 Do not know<br>99 No response

note_card	Vaccination Card	
card *(required)*	Do you have [child's name]'s immunization record card?<br>May I see it please?	1 If observed<br>2 Not observed<br>3 It does not have<br>98 Do not know<br>99 No response
date_bcg1_e *(required)*	BCG (Tuberculosis) Date Status First Dose<br>*Question relevant when: selected($(card} , '1')*	1 Complete date<br>2 Month and year only<br>98 Not legible<br>99 Not registered
date_bcg 1 {required}	Date BCG (Tuberculosis) First Dose<br>*Question relevant when: selected(${date bcg1 e} , '1') or selected($(date bcg1_e} , '2)*	
date_spr1_e *(required)*	Date status MMR (Measles, Rubella and Mumps) First Dose Question<br>*relevant when: selected(${card} , '1')*	1 Complete date<br>2 Month and year only<br>98 Not legible<br>99 Not registered
date_spr1 *(required)*	Date MMR (Measles, Rubella and Mumps) or MMR First Dose<br>*Question relevant when: selected($(date_spr1_e} , 'I) or selected($(date_spr1_e} , '2')*	
date_spr2_e *(required)*	Date Status MMR (Measles, Rubella and Mumps) Second Dose<br>*Question relevant when: selected($(card} , '1)*	1 Complete date<br>2 Month and year only<br>98 Not legible<br>99 Not registered
date_spr2 *(required)*	Date MMR (Measles, Rubella and Mumps) or MMR Second Dose<br>*Question relevant when: selected(${{date_spr2 e} , '1') or selected($(date_spr2 e} , '2')*	
covid_no_vaccine	At any time since the start of the pandemic, that is, March 2020 to date, did you avoid, postpone, or seek but not receive vaccination services for any of your children?	1 Yes<br>0 No<br>98 Do not know<br>99 No response
covid_no_vaccine_because *(required)*	Why did you avoid, postpone or seek and not receive vaccination services?<br><br>*Check all that apply*<br>*Question relevant when: selected($(covid_no_vaccine) , '1)*	1 The health unit was closed<br>2 The health unit was not acceptingpatients at that time. moment<br>3 No appointments were available<br>4 I went to the unit, but I did not attended<br>5 I was afraid of contagion from the COVID-19<br>6 I preferred to wait for the COVID-19 infections<br>7 I had to take care of some household member<br>8 Had difficulty in transportation to unity<br>9 I had symptoms of COVID-19 and had to stay at home<br>10 I had financial difficulties to pay for the service<br>88 Other (specify)
other_covid_no_vaccine *(required)*	Other reason for avoiding or postponing vaccination services<br>*Question relevant when: selected(${covid_no_vaccine_because) , '88')*	
parasites_note	Antiparasitics	
parasites (required)	In the past 6 months, has [child's name] been given any medication for intestinal parasites?	1 Yes<br>0 No<br>98 Do not know<br>99 No response
n parasites *(required)*	Show image<br>How many times have you given this medicine for intestinal parasites to [child's name] in the last 12 months?<br><br>Show image.<br>*don't know 98 No answer 99*<br>Question relevant when: selected(${parasites} , '1')	

diarrhea_notes	**Diarrhea**			
diarrhea (required)	In the last 2 weeks, did [child's name] have diarrhea?		1<br>2<br>98<br>99	Yes<br>No<br>Do not know<br>No response
advice (required)	On that occasion did you seek advice or treatment for diarrhea from any source?<br>*Question relevant when: selected(${diarrhea} , '1')*		1<br>2<br>98<br>99	Yes<br>No<br>Do not know<br>No response
where_advice (required)	Where did you seek counseling or treatment?<br>Question relevant when: selected(${advice} , '1')<br>-		1<br>2<br>3<br>4<br>5<br>6<br>7<br>8<br>9<br>10<br>11<br>12<br>13<br>88<br>98<br>99	Public hospital<br>Public clinic/public health center<br>Public mobile clinic<br>Other public facility<br>Private hospital<br>Private clinic/private health center<br>Private medical room<br>Private mobile clinic<br>Other private medical facility<br>Pharmacy<br>Community health promoter<br>Medical personnel<br>Traditional healer<br>Other<br>Don't know<br>No response
serum_polvo (required)	Were you given a drink or oral saline at any time since the onset of diarrhea?<br>*Question relevant when: selected(${diarrhea} , '1')*		1<br>2<br>98<br>99	Yes<br>No<br>Do not know<br>No response
fluid (required)	Were you given a drink or home-made liquid or fluid recommended by health personnel at any time since the onset of diarrhea?		1<br>2<br>98<br>99	Yes<br>No<br>Do not know<br>No response
diar_1_note	What else was [child's name] given to treat diarrhea?<br>I am going to mention some other common treatments and you answer me whether or not you gave it to [child's name] in his last episode of diarrhea.			
antibiotic_diarrhea (required)	Antibiotic<br>*Question relevant when: selected($(diarrhea} , '1')*		1<br>2<br>98<br>99	Yes<br>No<br>Do not know<br>No response
antimotility_agent (required)	Antimotility agent (iodochlorine, enteroguanil, intestinomycin, pepto bismol)<br>*Question relevant when: selected($(diarrhea} , '1')*		1<br>2<br>98<br>99	Yes<br>No<br>Do not know<br>No response
zinc_diarrhea (required)	Zinc. Show image<br>*Question relevant when: selected($(diarrhea} , '1')*		1<br>2<br>98<br>99	Yes<br>No<br>Do not know<br>No response
home_remedy (required)	Home remedy/herbal medicine<br>*Question relevant when: selected($(diarrhea} , '1')*		1<br>2<br>98<br>99	Yes<br>No<br>Do not know<br>No response

other_diarrhea (required)	Other<br>Question relevant when: selected($(diarrhea} , '1')		1<br>2<br>98<br>99	Yes<br>No<br>Do not know<br>No response
services_covid_children	**Child's use of health services during the pandemic**			
covid_avoid_serv_children (required)	At any time since the start of the pandemic, i.e., March 2020 to date, did you avoid, delay or seek but not receive medical care for any of your children from any of the following services?<br>*Check all that apply*		1<br>2<br>3<br>4<br>0<br>88	Well-child check-up<br>Vaccination<br>Diarrhea care<br>Respiratory illness care<br>None of the above<br>Other (specify)
other_covid_avoid_serving_children (required)	Other (specify)<br>*Question relevant when: selected($(covid_avoid_serv_children} , '88)*			
covid_avoid_serving_children_because (required)	Why did you avoid, postpone or seek but not receive medical care for those services?<br>*Check all that apply*<br>*Question relevant when: selected(${covid_avoid_serv_ninos} , '1)*		1<br>2<br>3<br>4<br>5<br>6<br>7<br>8<br>9<br>10<br>88	The health unit was closed<br>The health unit was not accepting patients at that time<br>No appointments were available<br>I went to the unit, but they would not see me<br>I was afraid of COVID-19 infection<br>I preferred to wait for COVID-19 infections to go down.<br>I had to take care of a household member<br>I had difficulty transporting myself to the unit<br>Had symptoms of COVID-19 and had to stay at home<br>Had financial difficulty paying for the service<br>Other (specify)
atn_maternal_note	**Antenatal care**			
atencion_pre (required)	When you became pregnant with [child's name] , were you receiving prenatal care?		1<br>2<br>98<br>99	Yes<br>No<br>Do not know<br>No response
who_consult (required)	Who did you consult?<br>*Select who consulted most of the time.*<br>*Question relevant when: selected(${atencion_pre) , '1')*		1<br>2<br>3<br>4<br>5<br>6<br>88<br>98<br>99	Physician<br>Nurse Practitioner<br>Auxiliary Nurse<br>Midwife<br>Community Health Promoter<br>Traditional Healer<br>Other<br>Don't know<br>No response

weeks (required)	How many weeks pregnant were you when you first received prenatal care for this pregnancy?<br><br>*If months are mentioned, convert to weeks by multiplying months by 4. If not sure, write 98. If no answer, write 99. Question relevant when: selected(${atention_pre} , '1')*	
n_consultas *(required)*	How many times did you receive prenatal care during this pregnancy?<br>*Question relevant when: selected($(atencion_pre} , 'l)*	1 One<br>2 Two<br>3 Three<br>4 Four<br>5 Five<br>6 Six or more<br>98 Do not know<br>99 No response
apn_mod_attention (required)	Do you use any of the following care modalities for at least one of your prenatal care?<br><br>*Check all that apply.*<br>*Question relevant when: selected($(atencion_pre} , '1')*	1 In-person consultation at the unit of health<br>2 Telephone consultation<br>3 WhatsApp Consultation<br>4 Video consultation by platform digital (Zoom, Teams or other application)<br>5 Home visit<br>6 Care brigades<br>88 Other (specify)
other_apn_mod_attention *(required)*	Another modality of attention<br>*Question relevant when: selected($(apn_mod_attention} , '88')*	

obtuvo_consent > encuesta_mef > hh_elegible > Add information for each woman aged 15 to 49 living in this household) > obtuvo_consent2 > Menores 5 General > Menores de 5 años (1) > apn group

Group delevent when. selected($(otencion_9re} , '1')

apnproup_note	As part of your prenatal care, are any of the following procedures performed on you as part of your prenatal care?<br>at least once when she was pregnant with [child's name] ?	
apnproup_label	*Procedure*	1 Yes<br>0 No<br>98 Do not know<br>99 No response
apn_cpeso (required)	…weighed?	1 Yes<br>0 No<br>98 Do not know<br>99 No response
apn_cpresion (required)	...your blood pressure (blood pressure) was taken? *Gesture with your arm.*	1 Yes<br>0 No<br>98 Do not know<br>99 No response
apn_c_eorina *(required)*	...did you submit a urine sample?	1 Yes<br>0 No<br>98 Do not know<br>99 No response
apn_c_esangre *(required)*	...did you submit a blood sample?	1 Yes<br>0 No<br>98 Do not know<br>99 No response
apn_c_futerino *(required)*	...they measured the size of your belly/belly/abdomen with a tape, i.e. they measured your fundus*.<br>*Gesture on the abdomen*	1 Yes<br>0 No<br>98 Do not know<br>99 No response
apn_c_ultrason (required)	...did you have an ultrasound or ultra?	1 Yes<br>0 No<br>98 Do not know<br>99 No response
apn_c_fcf (required)	...the doctor or nurse did a test to listen to your child's heartbeat while he/she was still in the womb?	1 Yes<br>0 No<br>98 Do not know<br>99 No response

apn_c_psida *(required)*	...were you offered a test to detect the HIV/AIDS virus?	1 Yes<br>0 No<br>98 Do not know<br>99 No response
apn_cpf *(required)*	.. ...are you advised about contraception after childbirth?	1 Yes<br>0 No<br>98 Do not know<br>99 No response
ppc_note	Childbirth and puerperium	
parto_attendance *(required)*	Who attended the birth of [child's name] ?	1 Physician<br>2 Nurse Practitioner<br>3 Auxiliary Nurse<br>4 Midwife<br>5 Community Health Promoter<br>6 Traditional Healer<br>88 Another<br>98 Do not know<br>99 No response
birth_place *(required)*	Where did you give birth to [child's name] ?	1 Your home<br>2 Someone else's house<br>3 Government Hospital<br>4 Public Clinic, Public CS<br>5 Public Medical Room<br>6 Other Public Establishment<br>7 Private Hospital<br>8 Private/Private Clinic,<br>9 Private Medical Room<br>10 Other private establishment<br>88 Another<br>98 Do not know<br>99 No response
ost *(required)* test	One week after delivery, did a health care provider (doctor, nurse or promoter) examine you for postpartum care?<br><br>If yes, did this occur at home or in a health care facility?	1 Yes, at home<br>2 Yes, in a medical facility<br>3 No<br>98 Do not know<br>99 No response
ppc_mod_attention {required}	Do you use any of the following modalities of care for postpartum care?<br><br>*Check all* that *apply.*	1 In-person consultation at the unit of health<br>2 Telephone consultation<br>3 WhatsApp Consultation<br>4 Video consultation by platform digital (Zoom, Teams or otherapplication)<br>5 Home visit<br>6 Care brigades<br>88 Other (specify)
serv_pregnancy_avoid *(required)*	At any time during your pregnancy with [child's name] did you avoid, postpone, or seek but not receive medical care for any of the following services?<br><br>*Check all that apply.*<br>*Question relevant when: $(date_nino} >-- date('2020-03-31')*	1 Prenatal care<br>2 Delivery care in a unit of health<br>3 Postpartum care for you<br>4 Newborn care<br>0 None of the above<br>98 Do not know<br>99 No response
serv_pregnancy_avoid_because *(required)*	Why did you avoid or postpone medical care for those services? Check all that apply<br>*Question relevant when: selected($(serv_pregnancy_avoid} , '1') or selected(${serv_pregnancy_avoid} , '2') or selected(${serv_pregnancy_avoid} , '3) or selected($(serv_pregnancy_avoid} , '4')*	1 The health unit was closed<br>2 The health unit was not acceptingpatients at that time. moment<br>3 No appointments were available

		4 I went to the unit, but I did not attended<br>5 I was afraid of contagion from the COVID-19
		6 I preferred to wait for the COVID-19 infections<br>7 I had to take care of some household member<br>8 Had difficulty in transportation to unity<br>9 I had symptoms of COVID-19 and had to stay at home<br>10 I had financial difficulties to pay for the service<br>88 Other (specify)
control_nino	**Well-child check-up**	
nino_6months *(required)*	In the last 6 months, did your child [child's name] receive any well-child checkups?	1 Yes<br>0 No<br>98 Do not know<br>99 No response
child_6months_where *(required)*	In which unit did your child receive this check-up?<br>*Question relevant when: Selected($(nino_6months) , '1')*	1 Public hospital<br>2 Public clinic/health center public<br>3 Public mobile clinic<br>4 Other public establishment<br>5 Private hospital<br>6 Private clinic/health center private<br>7 Private medical room<br>8 Private mobile clinic<br>9 Other medical facility private<br>10 Pharmacy<br>11 Community health promoter<br>12 Medical staff<br>13 Traditional healer<br>88 Another<br>98 Do not know
nino_6month_mod_attention *(required)*	Did you use any of the following care modalities for that well-child checkup?<br><br>*Check all that apply*<br>*Question relevant when: selected($(nino_6meses) , '1)*	1 In-person consultation at the unit of health<br>2 Telephone consultation<br>3 Whatsapp Consultation<br>4 Video consultation via digital platform (Zoom, Teams or other) application)<br>5 Home visit<br>6 Care brigades<br>88 Other (specify)
micro_note	Micronutrients	
mn_ac_useever *(required)*	Have you ever given [child's name] micronutrient powders or prodipitas?<br><br>*Show image.*	1 Yes<br>0 No<br>98 Do not know<br>99 No response
mn_ac_useyes *(required)*	For what reasons have you given micronutrient powders to your child'7?<br>*Question relevant when: selected(${mn_ac useever} , '1')*	1 A physician/nurse practitioner will prescribed<br>2 Gave them to him/her as a gift<br>3 Important for growth and child development<br>4 People in your community will recommended

		5 Because it helps to prevent/treat iron deficiency (anemia)<br>6 Because my son was diagnosed with anemia<br>88 Another reason, which one?
		98 Do not know
		99 No response
mn_ac_useyes_other	*Question relevant when: se1ected(${mn_ac useyes} , '88)*	
mn_ac_agestart	At what age in months did you start giving micronutrients?<br><br>*Enter 98 if don't know, 99 if no answer. If answered in years convert to months by multiplying by*<br><br>*Question relevant when: selected(${mn_ac_useever} , '1')*	
packages *(required)*	In the last 6 months, did you receive any sachets or packets of micronutrient powder (PRODIPITAS) for [child's name]?<br><br>*Show image of micronutrient sachets (PRODIPITAS) Question relevant when: selected(${mn_ac_useever} , '1')*	1 Yes<br>0 No<br>98 Do not know<br>99 No response
n_packages *(required)*	In the last 6 months, how many packages or envelopes have you received?<br><br>I would say that...<br>*Question relevant when: selected($(packages} , '1')*	1 1 a 10<br>2 11 a 20<br>3 21 a 30<br>4 31 a 40<br>5 41 a 50<br>6 51 a 60<br>7 More than 60<br>98 Do not know<br>99 No response
mn_ac_where *(required)*	Where did you receive the micronutrients?<br>*Question relevant when: selected($(packages} , 'l')*	1 Minsal establishment<br>2 Establishment of ISSS<br>3 Women's City<br>4 Pro-Family Clinic (ADS)<br>5 Clinic or private physician<br>6 ISBM<br>7 IPSFA<br>8 Pharmacy<br>9 Minsal Promoter<br>10 NGO promoter<br>88 Another<br>98 Do not know<br>99 No response
package_days	In the last 6 months, on how many days did [child's name] consume these packages? *Question relevant when: selected(${packages} , '1')*	
mn_tx_end *(required)*	In the last 6 months, did you finish giving [child's name] the full course of micronutrients?<br>*Question relevant when: selected(${packages} , '1')*	1 Yes<br>0 No<br>98 Do not know<br>99 No response
mn_tx_end_why *(required)*	Why didn't you finish the treatment?<br><br>*Check all of the above.*<br>*Question relevant when: selected(${mn_tx_end} , '0')*	1 I started recently (less than 2 months)<br>2 My child does not like them<br>3 Caused my child's allergy<br>4 My child got diarrhea,<br>5 My son didn't like it<br>6 The doctor told me to stop give<br>7 They are useless<br>8 My child does not need them<br>9 I had already taken enough<br>10 The treatment is very long<br>11 It is very laborious (difficult)<br>88 Another

mn_tx_vend *(required)*	Why did the treatment end?<br><br>*Check all* that *apply. Question relevant when: selected(${mn_tx_end} , 'I)*	1	It makes my child feel more comfortable and healthy
		2	Makes my child more comfortable strong
		3	Causes my child to gain weight
		4	Protect my child from diseases
		5	Prevents anemia/anemia deficiency iron in my child
		6	Improves growth and development of my child
		7	Because it gives you more energy
		8	Because it increases your appetite
		88	Another
mn_ac_visitmn *(required)*	During the last 6 months, did any health promoter visit you to talk about micronutrients?	1	Yes
		0	No
		98	Do not know
		99	No response
micro_note2	Usual Preparation **Micronutrients**<br>*Question relevant when: selected(${packages} , '1')*		
mn_ad_howoften *(required)*	How often do you usually give (or did you give when you had) micronutrients to your child [child's name]?<br>*Question relevant when: Selected($(packages) , 'I)*	1	Diary
		2	Alternate days (every other day)
		3	Twice a week
		4	Weekly
		5	Monthly
		6	Daily for 2 months and rest 4 months
		88	Another
		98	Do not know
		99	No response
mn_ad_foodused *(required)*	With what food do you usually prepare micronutrient powders when you give them to your child?<br><br>*Check up to 3 options*<br>*Question relevant when: selected($(packages} , 'I)*	1	Beans
		2	Rice
		3	Egg
		4	Ripe/banana
		5	Fruit
		6	Soup
		7	Fresh/juice
		88	Another
		98	Do not know
		99	No response
mn_ad_foodused_another_eng	What other food do you usually give your child micronutrients with?<br>*Question relevant when: selected($(mn_ad_foodused} , '88')*		
mn_ad_foodchange *(required)*	Do the micronutrient powders or Prodipitas produce any change to the food when you add them?<br>*Question relevant when: selected($(packages} , 'I)*	1	Yes
		0	No
		98	Do not know
		99	No response
mn_ad_foodchange_detail *(required)*	How does the food change?<br><br>*Check all of the above.*<br>*Question relevant when: selected(${mn_ad_toodchange} , '1')*	1	Color
		2	Taste
		3	Texture
		4	Smell
		88	Another
mn_ad_timetoprovide *(required)*	How long does it usually take to give the micronutrients to your child [child's name] once you have prepared it?<br>*Question relevant when: selected($(packages} , 'I)*	1	1 to 10 minutes
		2	11 to 20 minutes
		3	21 to 30 minutes
		4	31 to 40 minutes
		5	41 to 50 minutes
		6	51 to 60 minutes
		7	More than one hour
		98	Do not know
		99	No response
mn_ad_childpresent reparation *(required)*	Does [child's name] usually see when you add micronutrients to food?	1	Yes

	Question relevant when: selected(${packages} , 'I)	0 No<br>98 Do not know<br>99 No response
mn_ad_childreaction *(required)*	How does [child's name] usually accept food that has the micronutrients?<br>*Question relevant when: selected($(packages} , 'I)*	1 Very good<br>2 Well<br>3 Bad<br>4 Very bad<br>98 Do not know<br>99 No response
mn_ad_childfinishesfood *(required)*	How often does [child's name] finish food that has micronutrients?<br>*Question relevant when: selected(* $(packages, *'1')*	1 Always<br>2 Almost always<br>3 Sometimes<br>4 Never<br>98 Do not know<br>99 No response
note_changes	Perception of changes in your child's health<br>*Question relevant when: selected($(packages) , '1')*	
mn_ph_changeenergy *(required)*	Have you noticed any changes in [child's name]'s energy since starting micronutrients?<br>*Question relevant when: selected(${packages} , '1')*	1 Yes<br>0 No<br>98 Do not know<br>99 No response
mn_ph_changeenergy_detail *(required)*	What change have you noticed in [child's name]'s energy level?<br>*Question relevant when: selected(${mn h_changeenergy} , '1')*	1 More energy<br>2 Less energy
mn_ph_changeappetite *(required)*	Have you noticed any changes in your [child's name] child's appetite since starting micronutrients?<br>*Question relevant when: selected(${packages} , '1')*	1 Yes<br>0 No<br>98 Do not know<br>99 No response
mn_ph_changeappetite_detail *(required)*	What change have you noticed in your child's appetite level since starting micronutrients?<br>*Question relevant when: selected(${mn_ph_changeappetite} , '1')*	1 More appetite<br>2 Less appetite
mn_ph_general *(required)*	In general, how do you think your [child's name] child's health has changed since you started giving him/her micronutrients?<br>*Question relevant when: selected($(packages) , '1')*	1 Enhanced<br>2 Same (no change)<br>3 Worsened
mn_ac_useno *(required)*	For what reason(s) have you not given your child micronutrients?<br>*Question relevant when: selected(${mn_ac_useever} , '0)*	1 You have not heard of them<br>2 A doctor or nurse will not has indicated<br>3 Does not consider them important<br>4 They are not easy to obtain<br>5 It is time consuming to prepare them (it takes a lot of time).<br>6 The child rejects them<br>7 Difficult to remember every day<br>8 It is not clear how to prepare it<br>9 Does not mix easily with food<br>10 The child has problems digesting them<br>11 Because my child does not have anemia<br>12 Because my son doesn't need them<br>88 Another reason
generated_note_name_505	Micronutrients calendar	
calendar *(required)*	Have you been provided with a calendar like this'?<br>*Show calendar or sample image*	1 Yes<br>0 No<br>98 Do not know<br>99 No response
obs_calendary *(required)*	Could you show me the calendar?<br>*Question relevant when: selected($(calendario} , '1')*	1 Yes, observed<br>2 Not observed<br>3 It does not have<br>98 Do not know<br>99 No response
note_iron	Iron supplements	

mn_iron_received *(required)*	In the last 6 months, did [child's name] receive iron or ferrous sulfate for [child's name] ?	1 Yes<br>0 No<br>98 Do not know<br>99 No response
mn_hierro_when *(required)*	How many days ago did [child's name] receive iron or ferrous sulfate supplementation?	
	Enter 98 if don't know and 99 if no answer.<br>*Question relevant when: selected($(mn_iron_received) , '1')*	
mn_hierro_usage *(required)*	From the time you received it until today, how many days have you given [child's name] iron orferrous sulfate treatment?<br><br>*Enter 98 if you do not know and 99 if you do not answer.*<br>*Question relevant when: selected(${mn_iron_received } , '1')*	
cwb_appetite *(required)*	How would you rate [child's name]'s level of appetite?	1 Excellent<br>2 Very good<br>3 Good<br>4 Regular<br>5 Bad
cwb_energy *(required)*	How would you rate [child's name]'s activity or energy level?	1 Very active<br>2 Normal<br>3 Not very active
note_aliments	Child feeding<br><br>Now I would like to ask you about the liquids or food that [child's name] has ingested on the day of [child's name].<br>I am interested to know if your son consumed the product I mentioned, including whether itwas combined with other foods.	
	obtuvo_consent > encuesta_mef > hh_elegible > Add information for each woman aged 15 to 49 living in this dwelling) > obtuvo_consent2 > Menores 5 General > Menores de 5 years (1) > alimentacion_1	
note_ali_1	Did [son_name] drink or eat yesterday?	
note_ali_1_label	*Food or beverage*	1 Yes<br>0 No<br>98 Do not know<br>99 No response
cwb_breastfed *(required)*	Breast milk	1 Yes<br>0 No<br>98 Do not know<br>99 No response
cwb_water *(required)*	Water	1 Yes<br>0 No<br>98 Do not know<br>99 No response
cwb_coffeetea *(required)*	Coffee or Tea	1 Yes<br>0 No<br>98 Do not know<br>99 No response
cwb_juice *(required)*	Juices	1 Yes<br>0 No<br>98 Do not know<br>99 No response
cwb_milkpowder *(required)*	Milk (fresh or powdered)	1 Yes<br>0 No<br>98 Do not know<br>99 No response
cwb_infantformula *(required)*	Baby milk or formula	1 Yes<br>0 No<br>98 Do not know<br>99 No response
cwb_yogurt *(requified)*	Cheese, yogurt or other food made from milk	1 Yes<br>0 No<br>98 Do not know<br>99 No response

obtained consent > mef survey > eligible hh > Add information for each woman 15 to 49 years old living in this household) > obtained consent2 > Under 5 General > Under 5 General > Under 5General > Under 5 General > Under 5 General > Under 5 General > Under 5 General > Under 5 General > Under 5 General > Under 5 General > Under 5 General > Under 5 General > Under 5 General > Under 5 General

5 years (1) > alimentacion_2

note_ali_2	Did [son_name] drink or eat yesterday?		
note_ali_2_label	*Food or beverage*	1	Yes
		0	No
		98	Do not know
		99	No response
cwb_fortifiedfood *(required)*	Fortified baby food (e.g., Cerelac and Nestum)	1	Yes
		0	No
		98	Do not know
		99	No response
cwb_incaparin *(required)*	Incaparin	1	Yes
		0	No
		98	Do not know
		99	No response
cwb_cereal *(required)*	Breakfast cereal	1	Yes
		0	No
		98	Do not know
		99	No response
cwb_breadrice *(required)*	Bread, rice, noodles, oatmeal and other foods made from grains	1	Yes
		0	No
		98	Do not know
		99	No response
cwb_potatoes *(required)*	White potatoes, yucca or sweet potato	1	Yes
		0	No
		98	Do not know
		99	No response
cwb'yellowfruit *(required)*	Carrots, tomatoes, melon or any other orange or yellow colored fruit or vegetable in the core	1	Yes
		0	No
		98	Do not know
		99	No response
cwb_greenveggies *(required)*	Any dark green leafy vegetable (e.g., chipilin, blackberry, spinach, chaya, or any dark green leafy vegetable).	1	Yes
		0	No
		98	Do not know
		99	No response
cwb_mangopapa *(required)*	Ripe mangos or papayas	1	Yes
		0	No
		98	Do not know
		99	No response

obtained consent > mef survey > eligible hh > Add information for each woman 15 to 49 years old living in this household (1) > obtained consent2 > Under 5 General > Under 5 General > Under 5General > Under 5 General > Under 5 General > Under 5 General > Under 5 General > Under 5 General > Under 5 General > Under 5 General > Under 5 General > Under 5 General > Under 5 General > Under 5 General

5 years (1) > food 3

note_ali_3	Did [son_name] drink or eat yesterday?		
note_ali_3_label	*Food or beverage*	1	Yes
		0	No
		98	Do not know
		99	No response
cwb_meat *(required)*	Any type of meat, such as beef, pork, goat, chicken, or duck	1	Yes
		0	No
		98	Do not know
		9 9	No response
cwb_eggs *(required)*	Eggs	1	Yes
		0	No
		98	Do not know
		99	No response
cwb_fishseafood	Fresh or dried fish or shellfish	1	Yes
		0	No
		98	Do not know
		99	No response
cwb_beans *(required)*	Beans or lentils	1	Yes
		0	No

		98 Do not know<br>99 No response
cwb_nuts *(required)*	Any food prepared with nuts or dried fruit, such as peanuts or cashews	1 Yes<br>0 No<br>98 Do not know<br>99 No response
anemia_knowledge	Knowledge of anemia	

obtuvo_consent > encuesta_mef > hh_elegible > Add information for each woman aged 15 to 49 living in this household (1) > obtuvo_consent2 > Under 5 General > Under 5 years (1) >cono_anemia

an_c_heardanemia *(required)*	Have you heard of anemia?	1 Yes<br>0 No
an_c_heardirondef *(required)*	Have you ever heard of iron deficiency or iron deficiency?	1 Yes<br>0 No
an_c_anemiacauses *(required)*	What are the causes of anemia?<br><br>*DO NOT READ ANY OPTIONS. Check all the ones Imentioned*	1 Lack of iron in the diet<br>2 disease or infection (parasitic infection, other infection such as HIV/AIDS)<br>3 Malaria<br>4 Intestinal parasites<br>5 Lack of breastfeeding<br>6 Heavy bleeding during menstruation<br>101 Dengue, chicungunya, zika<br>100 Post-operative bleeding<br>88 Other (specify)<br>98 Don't know<br>99 No answer
other_cause_anemia *(required)*	Other cause of anemia, specify<br>*Question relevant when: selected(${{an_c_anemiacauses} , '88')*	
an_c_anemiaproblem *(required)*	How much do you consider anemia to be a health problem?<br><br>*READ OPTIONS*	1 Not a problem<br>2 It is a minor problem<br>3 It is a moderate problem<br>4 It is a serious problem<br>98 Do not know<br>99 No response
an_c_anemiasymptoms *(required)*	What are the signs or symptoms of someone who is anemic?<br><br>*DO NOT READ ANY OPTIONS. Check all the ones I mentioned*	1 Lack of energy / Weakness<br>2 Pallor<br>3 Spoon-shaped sunkennails<br>4 Nails break easily<br>5 Desire to eat dirtor charcoal<br>6 Lack of appetite<br>7 More likely to get sick (less immune response to infection)<br>88 Other<br>98 Don't know<br>99 No answer
an_c_consequences *(required)*	What are the consequences of anemia/iron deficiency in young children?<br><br>*DO NOT READ ANY OPTIONS. Check all the ones I mentioned*	1 Delayed mental development<br>2 Delayed physical development or growth<br>0 None<br>88 Other<br>98 Don't know<br>99 No answer
an_c_consequences_another	What other consequence?<br>*Question relevant when: selected(${an_c_consequences} , '88')*	
an_cprevention *(required)*	How can anemia be prevented or treated?<br><br>*DO NOT READ ANY OPTIONS. Check all the ones I mentioned*	1 Breastfeeding<br>2 Feeding iron-rich food<br>3 Offering foods rich in vitamin C during or after meals.

		4 Providing iron supplements<br>5 Treating infections<br>6 Micronutrients in powder or Prodipitas<br>7 Ferrous Sulfate<br>88 Another<br>98 Do not know<br>99 No response
an_dx_chance *(required)*	On a scale of 0 to 10, what is the likelihood that your child has anemia? Where 0 is that you are sure he/she does not have anemia, and 10 is that you are sure he/she has anemia.	0 Not likely<br>1 1<br>2<br>3 3<br>4 4<br>5 Just as likely<br>6 6<br>7<br>8 8<br>9 9<br>10 Very likely<br>98 Do not know
anemia_test	**Anemia test**	
an_d_bloodtest6m *(required)*	Has your child [child's name] in the last 6 months had a blood sample or finger prick for anemia?	1 Yes<br>0 No<br>98 Do not know<br>99 No response
an d sensortest6m *(required)*	Has your child had his/her hemoglobin measured with a sensor for anemia in the last 6 months?<br><br>*Show image of the device*	1 Yes<br>0 No<br>98 Do not know<br>99 No response
an_d_where *(required)*	Where were you tested?<br>*Question relevant when: selected(${an_d_bloodtest6m} , 'l) or selected($(an_d_sensortest6m) , '1')*	1 Minsal establishment<br>2 Establishment of ISSS<br>3 Women's City<br>4 Pro-Family Clinic (ADS)<br>5 Clinic or private physician<br>6 ISBM<br>7 IPSFA<br>8 Pharmacy<br>9 Minsal Promoter<br>10 NGO promoter<br>88 Another<br>98 Do not know<br>99 No response
an_d_who *(required)*	Who performed the test'?<br>*Question relevant when: selected(${an_d_bloodtest6m) , 'l) or selected($(an d sensortest6m) , '1')*	1 Physician<br>2 Nurse Practitioner<br>3 Auxiliary Nurse<br>4 Panera<br>5 Community Health Promoter<br>6 Traditional Healer<br>88 Another<br>98 Do not know<br>99 No response
an_d_examexplain *(required))*	Did they explain what the test was for?<br>*Question relevant when: selected(${an_d_bloodtest6m} , 'l) or selected($(an_d_sensortest6m) , '1')*	1 Yes<br>0 No<br>98 Do not know<br>99 No response
an_d_knowresult *(required)*	Were you informed of the test result?<br>*Question relevant when: selected($(an_d_bloodtest6m) , 'l) or selected($(an_d_sensortest6m) , '1')*	1 Yes<br>0 No<br>98 Do not know<br>99 No response
an_d_examresult *(required)*	What was the result of the test?	1 Without anemia (healthy)

Field	Question	Answer
	Question relevant when: selected(${an_d_bloodtest6m} , '1) or selected($(en_d_sensortest6m) , '1')	2 At risk of anemia<br>3 With anemia

Field	Question	Answer
		98 Do not know<br>99 No response
an_d_prescribe *(required)*	Were you prescribed any supplements or medications for your child to prevent or treat anemia such as micronutrient powders (Prodipitas), ferrous sulfate or iron?<br>*Question relevant when: selected(${an_d_bloodtest6m} , '1') or selected(${an_d_sensortest6m} , '1')*	1 Yes<br>0 No<br>98 Do not know<br>99 No response
an_d_prescription *(required)*	What supplement or medication were you prescribed?<br>*Question relevant when: selected($(an_d_bloodtest6m} , '1') or selected(${an_d_sensortesł6m} , '1')*	1 Prodipitas (micronutrients)<br>2 Ferrous Sulfate<br>3 Iron<br>88 Another<br>98 Do not know<br>99 No response
an_d_nutritionadvice *(required)*	Were you given nutrition advice for your child?<br>*Question relevant when: selected($(an_d_bloodtest6m} , '1') or selected(${an d sensortesł6m} , '1')*	1 Yes<br>0 No<br>98 Do not know<br>99 No response
an_d_explainanemia *(required)*	Did they explain to you what anemia was?<br>*Question relevant when: selected(${an_d_bloodtest6m} , '1') or selected($(an_d_sensortest6m} , '1')*	1 Yes<br>0 No<br>98 Do not know<br>99 No response
an_d_explanemia_what *(required)*	What did they tell you about anemia?<br><br>*Interviewer: Do not mention the options. Select the appropriate ones according to the respondent's answer.*<br>*Question relevant when: selected(${an_d_bloodtest6m} , '1') or selected(${an_d_sensortest6m} , '1')*	1 It is iron deficiency in the blood<br>2 Corresponds to low levels of hemoglobin<br>3 It is caused by a diet low in animal foods (chicken, came, etc).<br>4 It is a disease that affects the child development<br>5 It is a disease that can be easily treat<br>88 Another answer, what*?<br>98 He does not remember what was explained to him
other_anemia_they_said *(required)*	Another answer<br>*Question relevant when: selected(${an_d_explanemia_what} , '88)*	
an_d_reference *(required)*	Were you referred to another health facility to treat your child's anemia?<br>*Question relevant when: selected(${an_d_bloodtest6m} , '1') or selected(${an_d_sensortest6m} , '1')*	1 Yes, to an intermediate ECOS<br>2 Yes, to a specialized ECOS<br>3 Yes, to another ECOS<br>4 Yes. to a hospital<br>88 Yes, to another health facility<br>0 No
other_ref_anemia	Other health facility, which one?<br>*Question relevant when: selected(${an_d_reference) , '88)*	
note_anemia_diag	Diagnosis of Anemia	
an_dx_ever *(required)*	Has your child ever been diagnosed with anemia2?	1 Yes<br>0 No<br>98 Do not know<br>99 No response
an_dx_age *(required)*	At what age in months were you diagnosed?<br><br>*Enter 98 if don't know 99 if no answer. If you answer in years, convert to months by multiplying by 12 months.*<br>*Question relevant when: selected(${an_dx ever) , 'I)*	
an_dx_where *(required)*	Where was the diagnosis made?<br>*Question relevant when: selected($(an_dx_ever} , 'I)*	1 Minsal establishment<br>2 Establishment of ISSS<br>3 Women's City<br>4 Pro-Family Clinic (ADS)<br>5 Clinic or private physician

	6	ISBM
	7	IPSFA
	8	Pharmacy

Field	Question	Answer		
			9	Minsal Promoter
			10	NGO promoter
			88	Another
			98	Do not know
			99	No response
an_dx_how *(required)*	How did you get the diagnosis of anemia? *Question relevant when: selected(${an_dx_ever} , '1')*		1	Laboratory tests
			2	Finger prick and analyzed blood
			3	They used a finger sensor
			88	Another
			98	Do not know
			99	No response

end_interview	Interview data	
obs1 *(required)*	Observations or comments on the interview	
num_visits *(required)*	Number of visits made	1 1<br>2 2<br>3 3
result *(required)*	Result of the visit	1 Full interview<br>2 Partially complete<br>3 Interview rejected<br>4 Absent household members<br>5 Unoccupied house<br>6 The address of the home<br>88 Another
resulted_another *(required)*	Specify<br>*Question relevant when: selected(${result} , '88)*	
